# Bromide Sequestration Using a Low-Cost Hybrid Composite of Calcium, Activated Carbon, Kaolin, and Starch

**DOI:** 10.3390/polym18202586

**Published:** 2026-10-20

**Authors:** Bandar A. Al-Mur, Mamdoh T. Jamal

**Affiliations:** 1 Department of Environment, Faculty of Environmental Sciences, King Abdulaziz University, Jeddah 21589, Saudi Arabia; 2 Department of Marine Biology, Faculty of Marine Sciences, King Abdulaziz University, Jeddah 21589, Saudi Arabia; mjamal1@kau.edu.sa

**Keywords:** bromide removal, porous mineral–carbon composite, kaolin, activated carbon, water treatment, adsorption kinetics, adsorption isotherms

## Abstract

Bromide in source waters promotes the formation of toxic brominated disinfection byproducts during chlorination and ozonation, necessitating efficient, affordable pretreatment materials. This study developed a low-cost hybrid composite, denoted Calcium–Activated Carbon–Kaolin–Starch (CAKS) composite, from kaolin, activated carbon, a calcium precursor and starch through starch-assisted consolidation followed by calcination under nitrogen. The synthesized material was characterized by Fourier-transform infrared spectroscopy, X-ray diffraction, nitrogen adsorption–desorption analysis, scanning electron microscopy, and energy-dispersive X-ray spectroscopy. CAKS exhibited a heterogeneous mesoporous structure with a Brunauer–Emmett–Teller surface area of 212.5 m^2^ g^−1^, a pore volume of 0.384 cm^3^ g^−1^, and an average pore diameter of 7.2 nm. Batch adsorption experiments showed that bromide uptake was strongly dependent on solution pH, adsorbent dosage, contact time, initial concentration, agitation speed, temperature, and the presence of competing ions. Maximum removal of approximately 97.3% was achieved at pH 5, close to the apparent point of zero charge of 5.5. Adsorption was rapid during the first 60 min and approached equilibrium after 90–120 min. The experimental adsorption capacity increased with increasing bromide concentration, reaching approximately 40.0 mg g^−1^ at an initial bromide concentration of 200 mg L^−1^. Equilibrium data were best described by the Langmuir model, which yielded a model-estimated maximum adsorption capacity of 81.30 mg g^−1^. Kinetic data were described best by the pseudo-second-order model, although film diffusion and intraparticle diffusion also contributed to the overall uptake process. Equilibrium data indicated heterogeneous adsorption behavior, while thermodynamic analysis suggested that bromide adsorption was thermodynamically favorable under the investigated conditions. Regeneration with 0.1 M Sodium hydroxide and reuse over five adsorption–desorption cycles was also evaluated. Results were obtained under controlled laboratory conditions.

## 1. Introduction

The increasing demand for safe drinking water has intensified concerns regarding contamination by inorganic and organic pollutants. Among these contaminants, bromide (Br^−^) has attracted considerable attention because of its widespread occurrence in groundwater, surface water, and seawater, originating from natural geological sources, agricultural activities, and industrial discharges [1]. Although bromide itself is relatively nontoxic at environmentally relevant concentrations, it can become problematic during water disinfection. In the presence of strong oxidants such as chlorine and ozone, bromide reacts with natural organic matter to produce brominated disinfection byproducts (Br-DBPs), including bromate, brominated trihalomethanes (THMs), and haloacetic acids (HAAs) [2,3,4]. These compounds are generally more toxic and carcinogenic than their chlorinated analogs, posing significant risks to human health [5,6].

Growing epidemiological and toxicological evidence has linked long-term exposure to brominated DBPs with adverse health effects, including bladder cancer, reproductive disorders, and developmental abnormalities [6,7]. Population-scale studies have demonstrated a strong association between brominated THMs and increased cancer incidence, while recent investigations have highlighted the elevated toxicity of highly brominated species [4,6]. Moreover, the ongoing discovery of new brominated byproducts further underscores the need to control bromide concentrations in source waters before disinfection [8]. Consequently, the removal of bromide has become a crucial strategy for minimizing Br-DBP formation and improving drinking-water safety.

Several technologies have been investigated for bromide removal, including membrane filtration, electrochemical treatment, chemical oxidation, and adsorption [1]. Membrane processes such as reverse osmosis and nano-filtration provide high removal efficiencies but are often constrained by membrane fouling, concentrate management, and high operational costs [9,10]. Electrochemical approaches offer promising results for controlling bromide and bromate, although substantial energy requirements limit their practical application [10]. Chemical oxidation may also transform bromide, but can simultaneously generate undesirable secondary products if operating conditions are not carefully controlled [1].

Among these technologies, adsorption has emerged as one of the most attractive methods because of its operational simplicity, economic feasibility, and adaptability to low-cost materials [11,12,13]. Activated carbon (AC) is widely used owing to its large surface area and porous structure; however, its adsorption performance toward specific anions is often limited by poor selectivity [13]. Kaolin, an abundant aluminosilicate clay, possesses a layered structure and ion-exchange properties that can be enhanced through thermal or chemical modification [14]. Calcium-based materials, particularly calcium oxide derived from calcium carbonate, exhibit a strong affinity for anionic species through precipitation and surface complexation mechanisms [15]. In addition, starch has been extensively employed as a biodegradable binder and pore-forming agent in composite fabrication, thereby improving porosity and mass transfer [16]. The rationale for combining these components is based on their complementary structural and surface functions rather than assuming that each component contributes through the same adsorption mechanism. Activated carbon provides a high-surface-area porous framework that enhances bromide accessibility and mass transfer.

In contrast, kaolin-derived aluminosilicate domains provide additional hydroxylated mineral surface sites whose charge is sensitive to solution pH. Starch primarily acts as a binder and pore-forming agent; its decomposition during calcination promotes the development of interconnected pores and improves access to internal adsorption sites rather than serving as the principal bromide adsorbent. Calcium-containing domains may provide additional surface sites for bromide association under favorable conditions. Thus, incorporation of these components into a single composite is intended to combine accessible porosity, efficient mass transfer, and chemically heterogeneous surface sites, which cannot be provided to the same extent by any one component alone.

Previous studies have demonstrated the applicability of kaolin-, starch-, and activated-carbon-based materials for the adsorption of divalent metal ions from aqueous solutions. Natural kaolinite has been investigated for the removal of Pb(II), Cd(II), Ni(II), and Cu(II), showing rapid uptake, with maximum adsorption occurring within approximately 30 min under the investigated conditions [17]. Modified starch-based adsorbents have also demonstrated considerable affinity toward divalent metal ions. Succinylated and oxidized corn starches were reported to adsorb Cu(II), Zn(II), Pb(II), and Cd(II), with maximum adsorption capacities of 0.534 mmol g^−1^ for Pb(II) on succinylated starch and 1.245 mmol g^−1^ for Cu(II) on oxidized starch [18]. More recently, a magnetic kaolin/starch/CuFe_2_O_4_/ZIF-9 composite exhibited a Ni(II) adsorption capacity of 45.01 mg g^−1^, demonstrating the potential of kaolin–starch hybrid structures for divalent-cation removal [19]. Similarly, a kaolinite–activated-carbon composite prepared from pulp waste and sugarcane bagasse achieved approximately 96.7% Pb(II) removal within 30 min under optimized conditions, illustrating the complementary contribution of mineral adsorption sites and a porous carbonaceous phase [20]. Collectively, these studies demonstrate that kaolin, starch-derived materials, and activated carbon can provide complementary surface functionality, ion-exchange capability, and porous transport pathways for metal-ion adsorption. However, most previous applications have focused on positively charged divalent metal ions, whereas the adsorption behavior of the monovalent Br^−^ anion is substantially different. Therefore, the application of such hybrid low-cost components specifically for bromide sequestration remains comparatively underexplored.

Nevertheless, despite considerable progress in adsorbent development for water treatment, there remains a lack of low-cost hybrid materials specifically designed for bromide sequestration. Most previous studies have focused on the removal of heavy metals and organic contaminants, while relatively few have addressed bromide, particularly in the context of suppressing DBP formation. Furthermore, many existing adsorbents require expensive precursors or complex synthesis procedures, limiting their practical large-scale implementation [11].

To address these significant research gaps, the study presents an innovative hybrid composite adsorbent (CAKS) composed of calcium, activated carbon, kaolin, and starch specifically designed to remove bromide from aqueous media. The composite is synthesized from inexpensive, readily available precursors via a simple starch-assisted consolidation method followed by calcination, thereby avoiding complicated and energy-intensive fabrication routes. This approach was designed to generate a porous structure containing diverse active sites, combining the adsorption capacity of activated carbon, the ion-exchange characteristics of metakaolin, and the potential contribution of calcium-containing domains to anion adsorption. The incorporation of starch as a pore-forming agent is expected to improve porosity and facilitate mass transfer, thereby enhancing adsorption kinetics and overall performance.

The primary objective of this study is to prepare and characterize the composite material (CAKS) and to evaluate its performance in the adsorption of bromide ions. The physicochemical properties of the developed material were systematically examined, and the effects of key operating conditions on bromide removal were investigated. Furthermore, adsorption behavior was assessed through kinetic, equilibrium, and thermodynamic analyses, and regeneration experiments were conducted to evaluate the material’s reusability and practical application potential.

## 2. Materials and Methods

### 2.1. Materials

Kaolin (Al_2_Si_2_O_5_(OH)_4_, particle size <45 µm), calcium chloride (CaCl_2_, ≥99%), soluble starch (analytical grade), and powdered activated carbon (PAC, from coconut shell, BET surface area 1100 m^2^/g) were obtained from Sigma-Aldrich (Burlington, MA, USA). Sodium bromide (NaBr, ≥99%), hydrochloric acid (HCl, 37%), and sodium hydroxide (NaOH, ≥98%) were used as received. Deionized water (18.2 MΩ·cm) was used for all solutions.

### 2.2. Synthesis of CAKS Composite

The CAKS composite was prepared using a starch consolidation method followed by calcination under an inert atmosphere. The starch consolidation technique is a well-established method in ceramic processing, where starch acts as both a binder and a pore-forming agent, creating a macroporous structure upon burnout [16]. In a typical synthesis, 40 g of kaolin, 10 g of CaCl_2_, and 5 g of powdered activated carbon were dry-mixed in a mortar for 30 min to obtain a homogeneous powder mixture. Separately, 10 g of soluble starch was dissolved in 50 mL of deionized water at 70 °C under continuous stirring. The warm starch solution was then added gradually to the dry powder mixture and kneaded into a uniform paste. The resulting paste was dried in an oven at 105 °C for 24 h. After drying, the solid was gently crushed and placed in a tube furnace for calcination at 750 °C for 3 h with a ramp rate of 5 °C/min under continuous nitrogen flow (100 mL/min) to prevent excessive oxidation of the activated carbon.

The calcination temperature of 750 °C was selected based on established literature, as this temperature is sufficient to achieve nearly complete dehydroxylation of kaolin to metakaolin while avoiding the transformation to other phases [21]. During thermal treatment, any hydrated CaCl_2_ is expected to undergo dehydration. Under the N_2_ atmosphere used in this study, direct conversion of CaCl_2_ to CaO was not assumed or experimentally confirmed. Following calcination and washing, calcium remained detectable in the composite; however, XRD did not conclusively identify a specific calcium-containing crystalline phase. Therefore, the calcined material is conservatively described as containing calcium-containing domains rather than assigning them specifically to CaO. The use of an inert atmosphere (nitrogen) during calcination is essential when activated carbon is present, as it prevents the combustion of carbon that would otherwise occur in air at elevated temperatures. The calcined product was washed three times with deionized water until the wash water reached pH 8, then dried again at 105 °C for 12 h. Finally, the composite was sieved to a particle size range of 150–300 µm and stored in a desiccator. The resulting material is denoted CAKS. This synthesis approach combines the pore-forming capability of starch, surface sites associated with calcined kaolin and calcium-containing domains, and the enhanced surface area from activated carbon, producing a novel composite tailored for anion removal applications.

The synthesis yield was calculated using the equation: Yield (%) = (m CAKS/m initial dry precursors) × 100, where the total initial dry precursor mass was 65 g comprising 40 g of kaolin, 10 g of calcium chloride (CaCl_2_), 5 g of activated carbon, and 10 g of starch and m CAKS represented the mass of the composite material after washing, drying, and sieving. The synthesis process yielded approximately 40 g of CAKS, corresponding to an isolated yield of about 60%. To assess reproducibility, three independent batches were prepared, and the results were expressed as the mean value and standard deviation; the yield was 60.0 ± 2.0%, indicating good batch-to-batch consistency.

### 2.3. Characterization of CAKS

The surface functional groups of the CAKS composite were identified using Fourier-transform infrared spectroscopy (FTIR) over the range of 400–4000 cm^−1^. FTIR was used to examine changes in oxygen-containing and mineral-associated functional groups before and after Br^−^ adsorption; however, it was not used to assign a specific calcium phase. The crystalline and poorly ordered phases were examined by X-ray diffraction (XRD) using a Bruker D8 Advance diffractometer (Bruker AXS GmbH, Karlsruhe, Germany) with Cu Kα radiation (λ = 1.5406 Å) over a 2θ range of 10–80°. No specific calcium phase was assigned because the available XRD data did not permit unambiguous discrimination among possible CaO, CaCO_3_, calcium silicate, or other calcium-containing phases. The textural properties, including specific surface area, pore volume, and pore size distribution, were analyzed by nitrogen adsorption–desorption isotherms at 77 K using a Micromeritics ASAP 2020 surface area (Micromeritics Instrument Corporation, Norcross, GA, USA) and porosity analyzer, with the Brunauer–Emmett–Teller (BET) method applied for surface area calculation and the Barrett–Joyner–Halenda (BJH) model for pore size distribution. The surface morphology and elemental distribution of the CAKS composite before and after Br^−^ adsorption were examined using a JEOL JSM-7800F (JEOL Ltd., Akishima, Tokyo, Japan) field-emission scanning electron microscope equipped with an energy-dispersive X-ray spectroscopy (SEM-EDX) system to determine the elemental composition. Spatial attribution of Ca- and Br-containing regions requires elemental mapping. Collectively, these characterization techniques provided comprehensive insights into the structural, textural, morphological, and surface chemical properties that govern the bromide adsorption mechanism of the CAKS composite.

### 2.4. Batch Adsorption Experiments for Bromide Removal

#### 2.4.1. Stock Solution Preparation

A 1000 mg L^−1^ bromide stock solution was prepared by dissolving 1.287 g of sodium bromide, NaBr, in deionized water and diluting the solution to 1 L. Working bromide solutions with initial concentrations ranging from 5 to 200 mg L^−1^ were prepared by appropriate dilution of the stock solution using deionized water.

#### 2.4.2. Analytical Method for Br^−^

The residual bromide concentration in the aqueous samples was determined using the phenol-red colorimetric method described in Standard Method 4500-Br^−^ B. The method is based on the oxidation of bromide ions by Chloramine-T, followed by the bromination of phenol red under controlled acidic conditions. The resulting brominated dye develops a reddish-to-violet color, whose intensity is proportional to the bromide concentration and can be measured spectrophotometrically at 590 nm. Maintaining the reaction pH and reaction time is essential for obtaining reproducible measurements.

Before analysis, samples collected from the adsorption experiments were separated from the CAKS particles by filtration. Samples containing bromide concentrations above the working range were diluted with deionized water. A series of bromide standards covering 0–0.80 mg L^−1^ was prepared from a standard potassium bromide solution and treated in the same manner as the samples.

For each determination, 25 mL of sample or standard solution was transferred into a clean flask. Subsequently, 2.0 mL of acetate buffer solution at pH 4.6–4.7 and 2.0 mL of phenol-red indicator solution were added. The reaction was initiated by adding 0.5 mL of freshly prepared Chloramine-T solution, with thorough mixing after each reagent addition. The mixture was allowed to react for exactly 20 min. The remaining oxidant was then quenched by adding 0.5 mL of 2 M sodium thiosulfate solution.

The absorbance was measured immediately at 590 nm against a reagent blank using a UV–visible spectrophotometer. Bromide concentrations were obtained from a calibration curve prepared by plotting absorbance against standard concentrations. The final concentrations of the diluted samples were corrected using the appropriate dilution factors. The reported detection limit of the method is approximately 0.10 mg Br^−^ L^−1^, although the instrument application study demonstrated reliable calibration down to 0.05 mg L^−1^, with a coefficient of determination of R^2^ = 0.9992 [22,23].

#### 2.4.3. Optimization of Adsorption Parameters

The effects of the main operating parameters on bromide adsorption by the CAKS composite were investigated systematically using a one-factor-at-a-time approach, in which the parameter of interest was varied. At the same time, the remaining experimental conditions were maintained constant. The effect of initial pH was investigated over the range of 2–10 using an initial Br^−^ concentration of 20 mg L^−1^, a solution volume (V) of 25 mL (0.025 L), an adsorbent mass (m) of 0.10 g, corresponding to an adsorbent dosage (m/V) of 4.0 g L^−1^, a temperature of 25 ± 2 °C, and a contact time of 180 min. Based on the pH experiments, pH 5 was selected for the subsequent adsorption experiments. The effect of initial Br^−^ concentration was then examined over the range of 5–200 mg L^−1^ under the selected conditions. The effect of adsorbent dosage was evaluated by varying the mass of CAKS from 0.05 to 1.5 g (corresponding to dosages of 2.0–60.0 g L^−1^) while keeping the other treatment conditions constant. The influence of temperature was investigated at 25, 30, 35, 40, 45, 50, and 55 °C using a temperature-controlled shaking water bath, whereas the effect of agitation speed was examined over the range of 50–250 rpm. Contact-time experiments were conducted until the adsorption equilibrium was reached. For each parameter investigated, only the selected variable was varied, while the remaining treatment conditions were held constant to ensure consistency and comparability across experiments (Table S1).

The equilibrium adsorption capacity (q_e_, mg g^−1^) and percentage removal (%) were calculated as:

(1) $$Adsorption\;capacit(q_{e}\frac{mg}{g})=\frac{\left(C_{o}-C_{e}\right)\times V}{m}$$

(2) %Removal=((Co−Ce)/Co)×100

where C_o_ and C_e_ (mg L^−1^) are the initial and equilibrium Br^−^ concentrations, V (L) is the solution volume, and m (g) is the mass of adsorbent. Reagent blanks (without adsorbent) and adsorbent blanks (without Br^−^) were included for baseline correction.

### 2.5. Point of Zero Charge (pH_PZC_)

The point of zero charge of the CAKS composite was determined using the pH-drift method. A series of 50 mL portions of 0.1 M NaCl solution was prepared, and the initial pH was adjusted over the range of 2–10 using 0.1 M HCl or 0.1 M NaOH. Subsequently, 0.1 g of the CAKS composite was added to each solution.

The suspensions were shaken at 200 rpm for 24 h at room temperature (25 ± 1 °C) to allow the system to reach equilibrium. The final pH of each suspension was then measured, and the difference between the final and initial pH values was calculated as ΔpH = pH_f_ − pH_i_. The ΔpH values were plotted against the initial pH, and the pH_PZC_ was identified at the point where ΔpH = 0. At this point, the CAKS surface carries no net electrical charge because proton uptake and release by the surface functional groups are balanced.

### 2.6. Adsorption Study

The adsorption rate and the possible rate-controlling steps involved in Br^−^ uptake by the CAKS composite were investigated using several kinetic models. During the kinetic experiments, the remaining Br^−^ concentration in solution was measured at predetermined contact times until no further significant change was observed. The experimental data were fitted to the pseudo-first-order, pseudo-second-order, intraparticle diffusion, and film diffusion models.

The pseudo-first-order model relates the adsorption rate to the number of vacant sites remaining on the adsorbent surface. In contrast, the pseudo-second-order model describes the adsorption rate in terms of the availability of active sites and adsorbate–surface interactions. The intraparticle diffusion model was applied to determine whether Br^−^ transport through the composite pores contributed to the overall adsorption rate. In addition, the film-diffusion model was used to examine the resistance associated with the transfer of Br^−^ ions from the bulk solution to the external surface of the adsorbent. The suitability of each model was evaluated using the coefficient of determination (R^2^) and the agreement between the calculated and experimentally measured adsorption capacities. The equations and corresponding parameters used for the kinetic analysis are listed in Table 1.

The equilibrium data were analyzed using the Langmuir, Freundlich, Temkin, and Dubinin–Radushkevich isotherm models. These models were selected because they represent different assumptions regarding the surface properties and adsorption behavior of the composite. The Langmuir model describes monolayer adsorption on a surface containing a finite number of equivalent sites. In contrast, the Freundlich model accounts for adsorption on a heterogeneous surface with sites of unequal adsorption energies. The Temkin model accounts for the effect of adsorbent–adsorbate interactions on the heat of adsorption, while the Dubinin–Radushkevich model provides information on the apparent adsorption energy and the possible nature of the uptake process. Model performance was assessed using the regression coefficients and the physical relevance of the calculated parameters. All isotherm equations and model constants are presented in Table 1. In addition to the regression coefficient, the agreement between the experimental adsorption capacities and the predictions of the Langmuir, Freundlich, Temkin, and Dubinin–Radushkevich models was evaluated using the root-mean-square error, average absolute relative error, and nonlinear chi-square error function. Lower root mean square error (RMSE), average absolute relative error (AARE), and the non-linear chi-square (χ^2^) error function values indicate closer agreement between the experimental and model-predicted data.

The thermodynamic characteristics of Br^−^ adsorption were examined at seven temperatures (25, 30, 35, 40, 45, 50, and 55 °C). For all temperature-dependent experiments, the initial Br^−^ concentration (C_o_) was 30 mg L^−1^, the solution volume (V) was 0.025 L, and the adsorbent mass (m) was 0.100 g, corresponding to an adsorbent dosage (m/V) of 4.00 g L^−1^. At each temperature, a dimensionless concentration-based equilibrium ratio, Kc, was calculated as K_c_ = (C_o_ − C_e_)/C_e_, where C_o_ and Ce are the initial and equilibrium Br^−^ concentrations, respectively. Based on the adsorption mass balance Equation (1), the ratio is equivalently related to the adsorption data by K_c_ = (q_e_/C_e_) × (m/V). Thus, C_o_, C_e_, q_e_, V, m, m/V, and K_c_ were cross-checked for mutual consistency at each investigated temperature.

It should be emphasized that this dimensionless concentration-based ratio is used here as an operational quantity for comparative Van’t Hoff analysis and is not considered a rigorous standard-state thermodynamic equilibrium constant. As discussed critically by Tran [24], rendering an adsorption ratio dimensionless does not, by itself, establish a rigorous standard thermodynamic equilibrium constant. Accordingly, the thermodynamic quantities derived from this ratio are reported here as apparent parameters rather than standard-state thermodynamic quantities.

The apparent Gibbs free-energy change, ΔG_app_, was then calculated to evaluate the thermodynamic favorability of Br^−^ adsorption under the investigated conditions [24]. The apparent enthalpy change, ΔH_app_, and apparent entropy change, ΔS_app_, were obtained from the slope and intercept of the Van’t Hoff plot of lnK_c_ against 1/T, respectively. These thermodynamic parameters were used to determine whether Br^−^ adsorption was endothermic or exothermic and to assess changes in the degree of order at the solid–solution interface. The equations applied in the thermodynamic analysis are also summarized in Table 1.

### 2.7. Regeneration Procedure and Reusability Assessment

The regeneration and reusability of the CAKS composite were evaluated over five consecutive adsorption–desorption cycles. After each adsorption cycle, the bromide-loaded adsorbent was separated from the solution by filtration and washed with deionized water to remove loosely bound bromide ions. Desorption was then carried out by treating the spent adsorbent with 0.1 M NaOH solution. Under alkaline conditions, deprotonation of the surface functional groups increases the negative surface charge, thereby weakening the electrostatic attraction between the composite and bromide ions and promoting their release into the solution. After desorption, the regenerated adsorbent was repeatedly washed with deionized water until the wash water reached approximately neutral pH. The material was subsequently dried at 60 °C and reused in the following adsorption cycle. The bromide removal efficiency was determined after each cycle to assess the stability and reusability of the composite.

After each regeneration step, the adsorption experiment was repeated under the same conditions as the initial adsorption cycle, including the initial bromide concentration, solution pH, adsorbent dosage, solution volume, contact time, agitation speed, and temperature. Thus, the observed changes in removal efficiency were attributed to repeated regeneration and reuse of the CAKS composite rather than to changes in the adsorption conditions.

### 2.8. Data Analysis

Experimental data were organized and analyzed using Microsoft Excel, and adsorption capacity and removal efficiency were calculated based on measured bromide concentrations. Kinetic and equilibrium data were fitted to corresponding adsorption models, and model performance was evaluated using the following parameters: the coefficient of determination (R^2^), root mean square error (RMSE), average absolute relative error (AARE), and the non-linear chi-square (χ^2^) error function. Furthermore, thermodynamic parameters were calculated from temperature-dependent equilibrium data; the Van’t Hoff equation was used to determine ΔH_app_, ΔSapp, and ΔGapp values, and graphical plotting and regression analyses were performed using Microsoft Excel. All batch adsorption experiments were conducted independently in triplicate (n = 3) under identical experimental conditions; the reported values represent the average of three independent measurements.

## 3. Results and Discussion

### 3.1. Structural and Textural

The CAKS composite was sieved to obtain particles in the 150–300 µm range, with a median diameter of approximately 225 µm. This particle size offers a suitable compromise between surface accessibility and hydraulic performance. It may reduce pressure loss in fixed-bed systems while maintaining sufficiently short diffusion pathways for bromide transport. During preparation, starch served as a temporary binder, consolidating the kaolin, powdered activated carbon, and calcium-containing components. Its decomposition during calcination at 750 °C generated interconnected pores and cavities within the mineral–carbon framework, resulting in a mechanically stable, heterogeneous granular structure [16].

The nitrogen adsorption–desorption isotherm of CAKS showed a Type IV profile with an H3 hysteresis loop, indicating a mainly mesoporous structure containing slit-shaped pores (Figure S1). This behavior is consistent with materials composed of plate-like mineral particles and irregular carbonaceous aggregates [25]. The composite exhibited a BET surface area of 212.5 m^2^ g^−1^, a total pore volume of 0.384 cm^3^ g^−1^, and an average pore diameter of 7.2 nm. Its surface area was considerably higher than that of raw kaolin because of the incorporation of powdered activated carbon and the formation of additional pores during starch burnout. The relatively wide mesoporous channels may improve the transport of hydrated Br^−^ ions through the granules and increase access to internal calcium-containing aluminosilicate and carbonaceous surface domains. Therefore, the novelty of the CAKS structure lies in combining a practical granular particle size with a hierarchical porous network within a single low-cost composite. This structural arrangement may support efficient mass transfer while preserving the hydraulic and mechanical properties required for batch and fixed-bed water-treatment applications.

### 3.2. Characterizations

**Figure 1:**
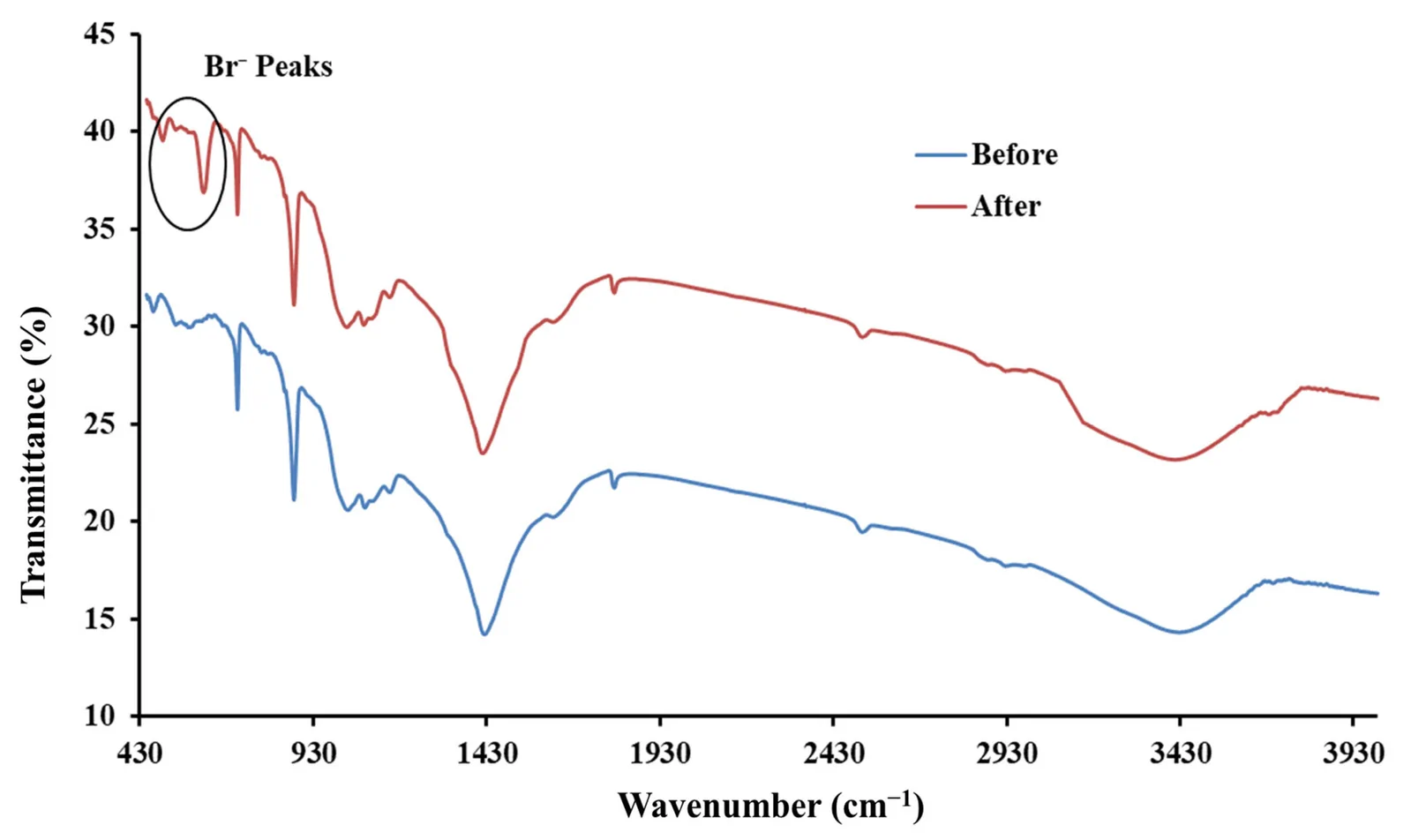
FTIR spectra of the CAKS composite before and after bromide adsorption.

The FTIR spectrum of the CAKS composite before bromide adsorption showed a broad band at approximately 3426 cm^−1^, assigned to O–H stretching of surface hydroxyl groups and adsorbed water (Figure 1 and Table S2). Bands near 1081 and 1030 cm^−1^ were attributed mainly to Si–O, Si–O–Al, and overlapping C–O vibrations from the meta kaolin and carbonaceous components. The bands at approximately 1425, 874, and 710 cm^−1^ were assigned to carbonate vibrations, whereas the bands near 575 and 471 cm^−1^ were associated with Al–O, Ca–O, and framework vibrations. After Br^−^ exposure, the spectrum showed small shifts and intensity changes in these regions (Figure 1). The O–H band shifted to about 3416–3420 cm^−1^ and became broader, suggesting modification of the hydrogen-bonding environment and interaction of bromide with protonated hydroxylated sites. Similar shifts in surface hydroxyl bands are commonly used as indirect evidence of anion adsorption on oxide and mineral surfaces [26,27]. In the fingerprint region, the bands near 1081 and 1030 cm^−1^ shifted to approximately 1075–1078 and 1023–1027 cm^−1^, respectively. These changes suggest that silanol, aluminol, Si–O–Al, and oxygen-containing carbon groups may contribute to bromide retention. The carbonate bands also showed small shifts, from 1425 to 1418–1422 cm^−1^, from 874 to 870–872 cm^−1^, and from 710 to 706–708 cm^−1^. Such changes may reflect alterations in carbonate-containing and other mineral surface domains; they do not identify a specific calcium phase. The low-wavenumber bands near 575 and 471 cm^−1^ were also expected to shift slightly, indicating possible modification of the local environment around metal–oxygen and framework sites. These changes support a mechanism involving electrostatic attraction, modification of surface hydration, and possible outer-sphere ion association. However, Br^−^ is a monatomic ion and does not produce a unique strong mid-infrared band. Therefore, FTIR provides only indirect evidence of bromide adsorption [28,29].

**Figure 2:**
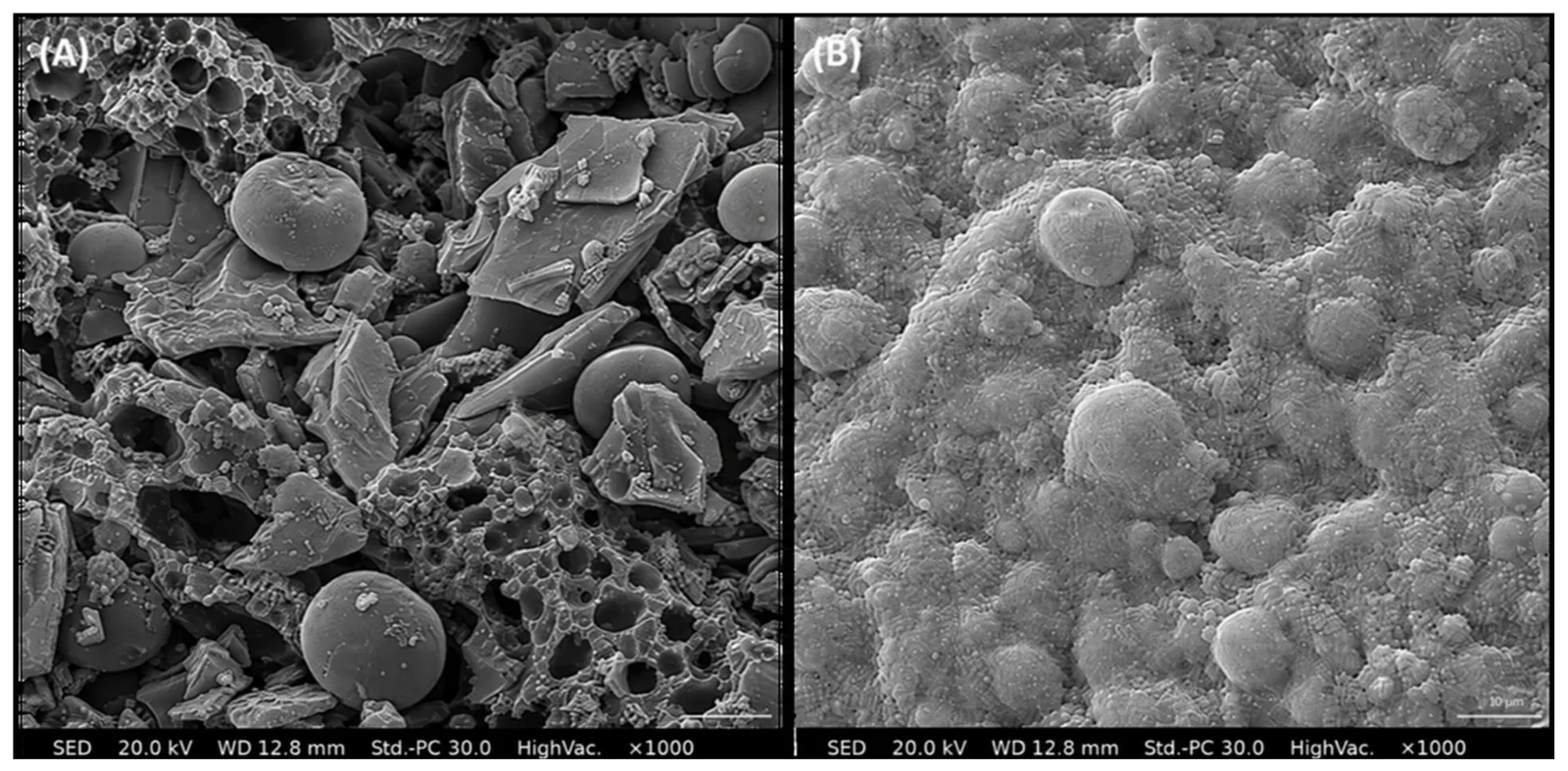
SEM micrographs of the CAKS composite (**A**) before and (**B**) after bromide adsorption at ×1000 magnification.

**Figure 3:**
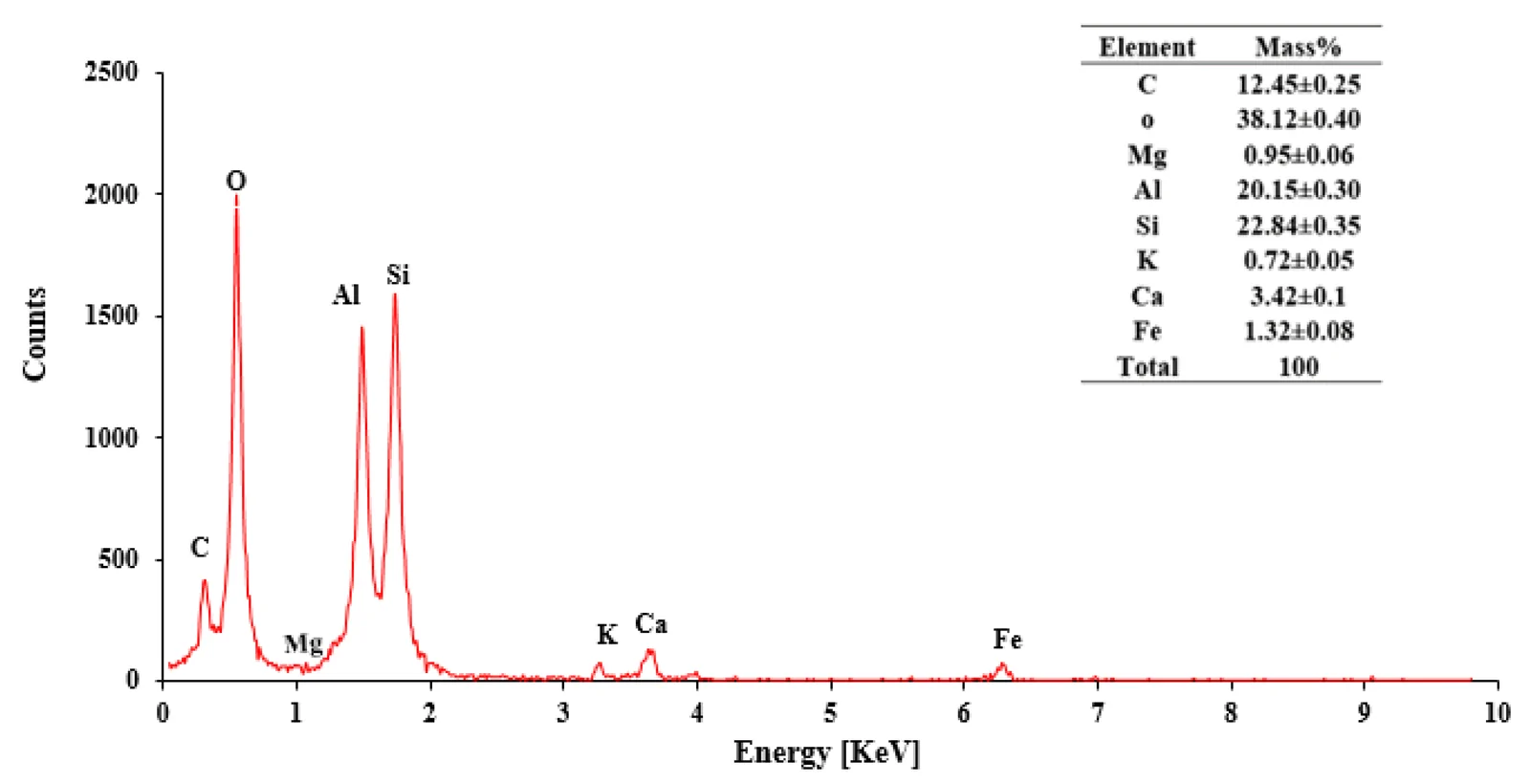
EDX spectrum and corresponding elemental composition of the CAKS composite material.

The SEM image before adsorption (Figure 2A) shows a heterogeneous porous surface containing plate-like kaolin particles, approximately spherical grains, and rough carbonaceous regions. The interconnected pores likely resulted from the decomposition of activated carbon and starch, thereby providing accessible adsorption sites [16]. After bromide adsorption (Figure 2B), the surface appeared denser and more agglomerated, with partially covered pores. This change suggests surface deposition and occupation of available cavities by adsorbed species. Similar morphological changes have been reported for bromide-loaded mineral and carbon-based adsorbents [28,29]. EDX analysis (Figure 3) confirmed the presence of the following elements: O (38.12%), Si (22.84%), Al (20.15%), C (12.45%), Ca (3.45%), Fe (1.32%), Mg (0.95%), and K (0.72%). The Si–Al–O composition confirms the kaolin framework, carbon indicates activated carbon and carbonized starch, and Ca confirms the presence of calcium in the composite but does not identify its chemical phase. EDX analysis confirmed the presence of Ca (3.45%) in the composite; however, EDX provides elemental rather than phase-specific information and therefore cannot determine whether the Ca is present as CaO, CaCO_3_, a calcium silicate, or another Ca-containing phase. The XRD pattern also did not provide sufficiently resolved and unambiguous reflections to distinguish among these possible calcium phases. Therefore, no specific calcium-containing crystalline phase is assigned in the present study. Accordingly, calcium is referred to throughout this study conservatively as calcium-containing or calcium-associated domains.

**Figure 4:**
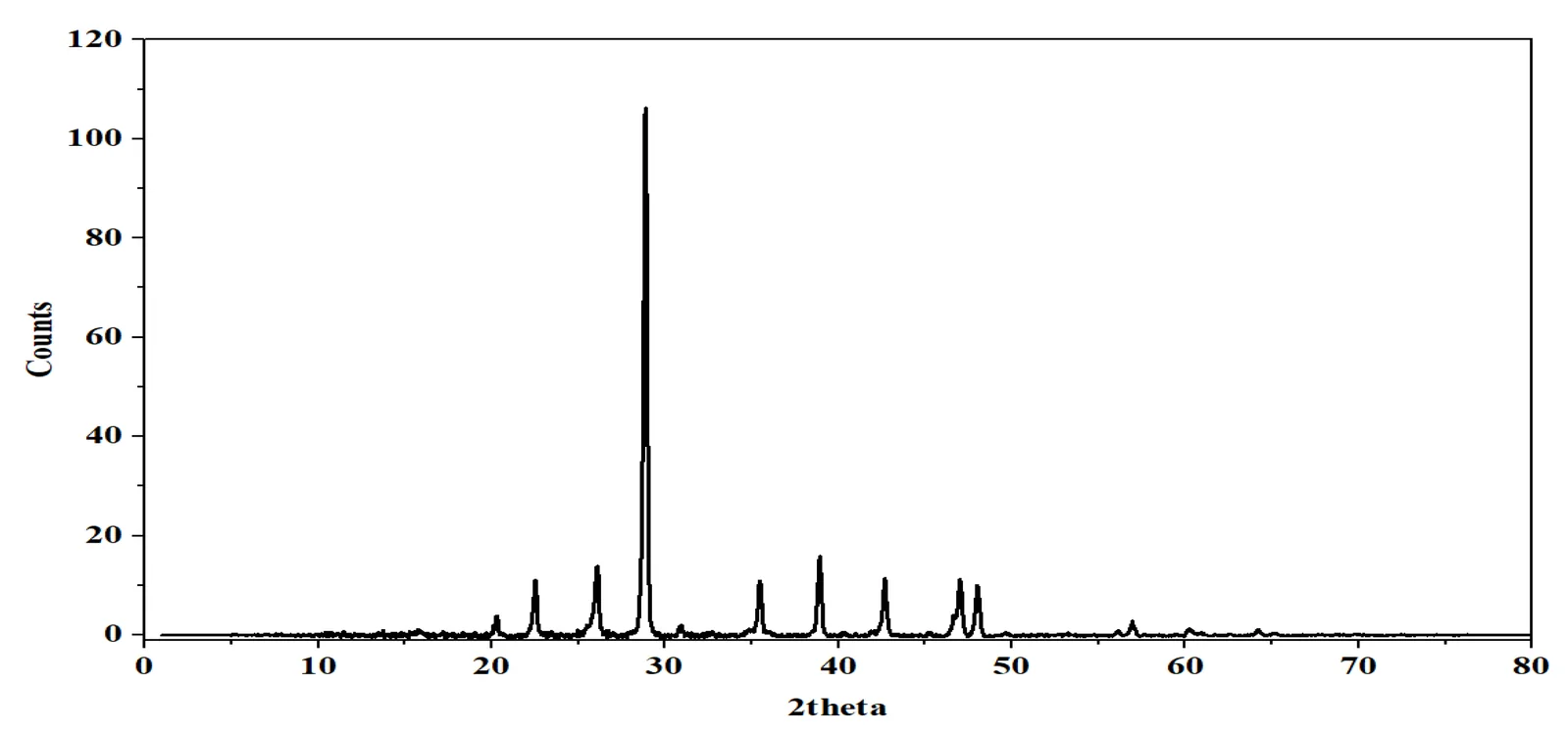
XRD pattern of the CAKS composite showing kaolin-derived aluminosilicate features and broad contributions from disordered carbonaceous material. No specific calcium-containing crystalline phase was conclusively identified from the present XRD pattern.

The XRD pattern indicates a heterogeneous composite containing kaolin-derived aluminosilicate phases and broad contributions from disordered carbonaceous material (Figure 4). Peaks near 20–26° are attributed mainly to kaolinite and starch, while the broad contributions around 24–26° and 43–44° are characteristic of disordered activated carbon [21,30]. Starch commonly exhibits reflections near 15°, 17°, 18°, and 23°, although its peaks can overlap with those of kaolin and carbon [31].

**Figure 5:**
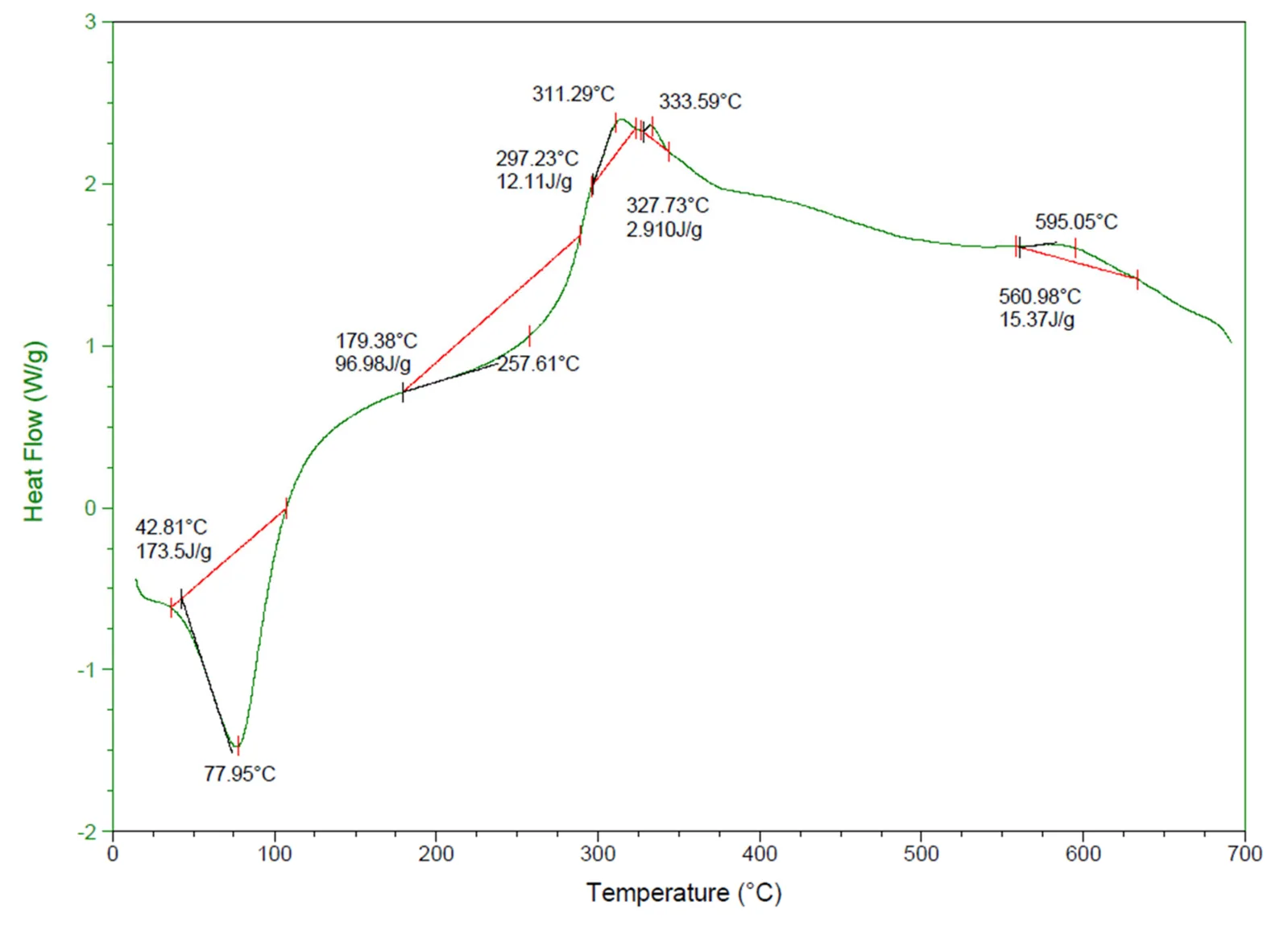

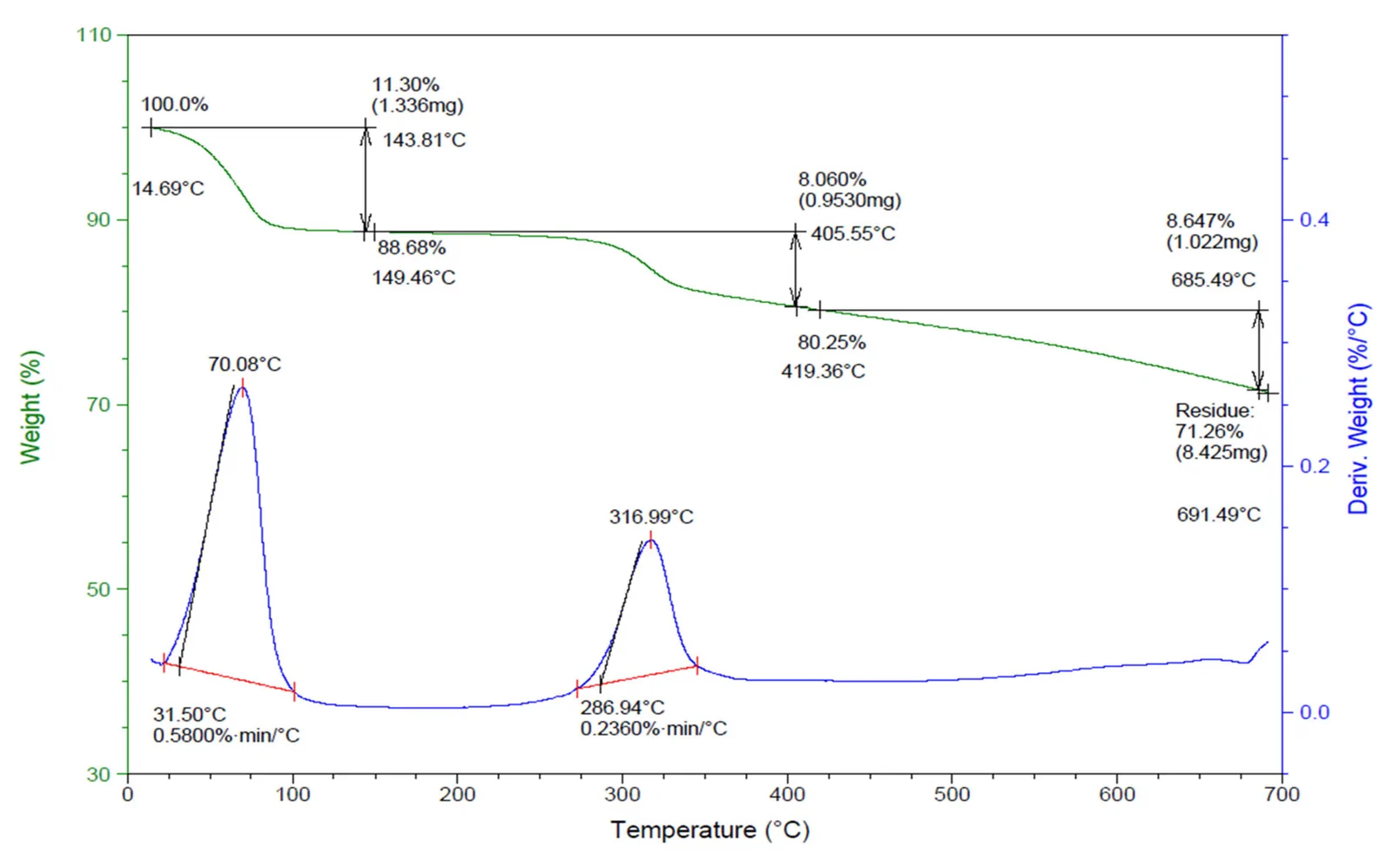
TGA–DSC thermograms of the CAKS composite.

The thermogravimetric analysis (TGA) curve, as shown in Figure 5, exhibited three main stages of mass loss. The first stage, approximately 11.30% up to 143.8 °C, is attributed to the evaporation of physically adsorbed water and dehydration of hygroscopic constituents, particularly CaCl_2_, kaolin, starch, and activated carbon. The strong differential scanning calorimetry (DSC) endothermic peak at approximately 77.95 °C supports the removal of moisture and weakly bound water. Hydrated CaCl_2_ can undergo sequential dehydration over this temperature region, depending on its hydration state. The second mass-loss stage, approximately 8.00% between 143.8 and 405.6 °C, is mainly associated with decomposition of starch and degradation of oxygen-containing functional groups in the carbonaceous fraction. The DSC events observed between approximately 257 and 334 °C, including peaks near 297.2, 311.3, 327.7, and 333.6 °C, correspond to overlapping thermal degradation, depolymerization, and carbonization processes. Starch commonly undergoes major thermal decomposition within this range, while activated carbon may lose residual surface oxygen groups. The third stage, approximately 8.65% between 405.6 and 685.4 °C, is attributed to further carbonaceous decomposition and dehydroxylation or structural rearrangement of the kaolin-derived aluminosilicate phase. The weak DSC events near 560.9 and 595.1 °C are consistent with transformations of clay-mineral hydroxyl groups and the progressive formation of a less-ordered metakaolin-type structure [21]. A high residual mass of approximately 71.26% at 691 °C indicates that the composite is predominantly inorganic and has good thermal stability.

### 3.3. Optimum Conditions

#### 3.3.1. Effect of Initial pH on Bromide Adsorption

**Figure 6:**
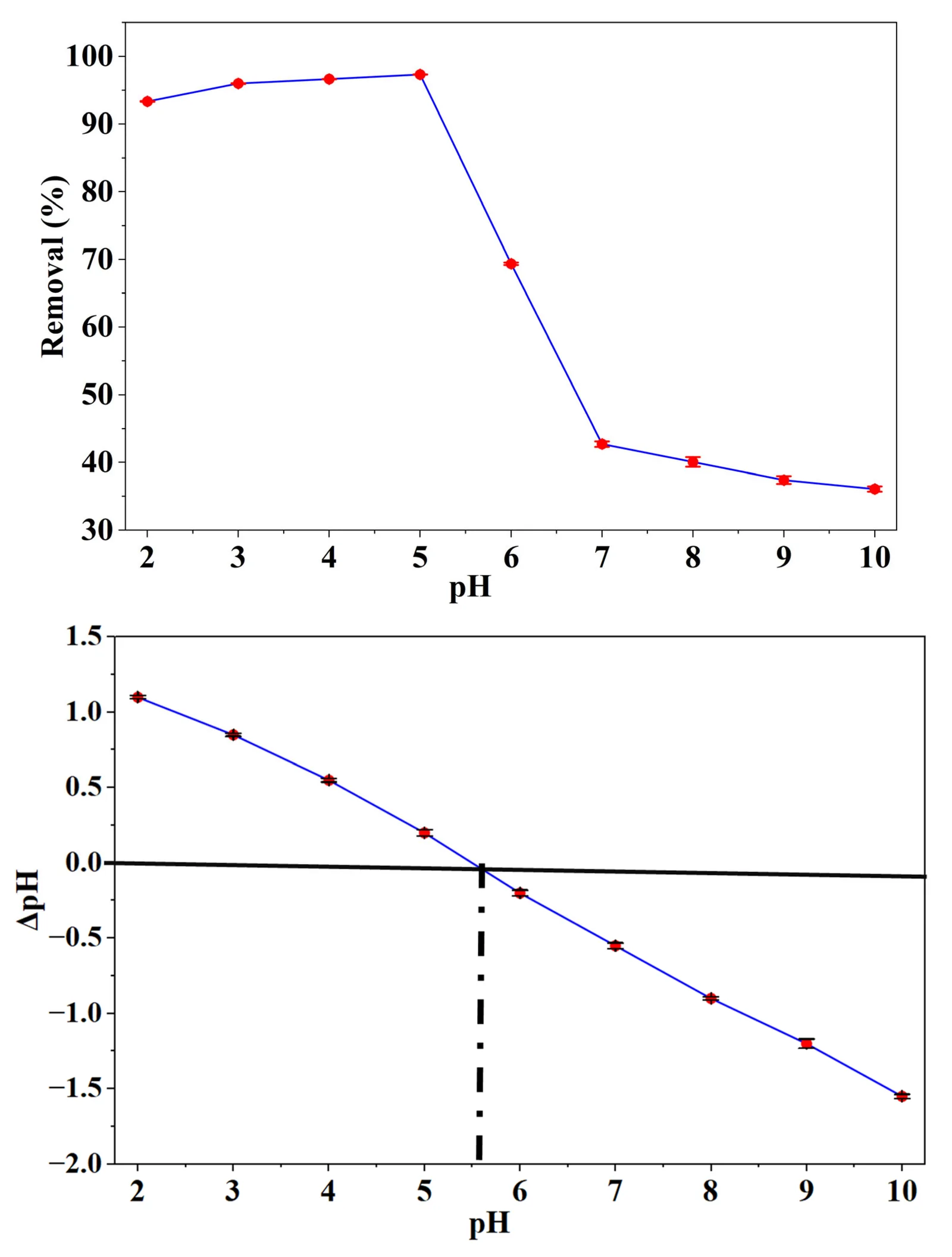
Determination of the point of zero charge (pH_p_zc) of the CAKS composite using the pH-drift method. The blue line represents the variation in ΔpH (pH_final_ − pH_initial_) with initial pH, while the black solid horizontal line indicates ΔpH = 0. The black dashed vertical line marks the estimated pH_p_zc of approximately 5.5.

The initial solution pH strongly influenced bromide adsorption by the CAKS composite. As shown in Figure 6, the Br^−^ removal efficiency increased from approximately 93.34% at pH 2 to 96.00% at pH 3 and 96.67% at pH 4, reaching a maximum of approximately 97.34% at pH 5. The removal efficiency then decreased to approximately 69.37% at pH 6 and declined further to approximately 42.73%, 40.06%, 37.40%, and 36.07% at pH 7, 8, 9, and 10, respectively. Therefore, pH 5 was selected as the optimum pH for Br^−^ adsorption.

The high removal efficiency under acidic conditions may be attributed to the protonation of functional groups on the adsorbent surface. At low pH, surface hydroxyl groups associated with activated carbon, kaolin, and calcium-containing phases may accept protons and form positively charged sites: ≡SOH + H^+^ ⇌ ≡SOH_2_^+^.

These positively charged sites can attract negatively charged Br^−^ ions through electrostatic interactions. The influence of pH on adsorption is commonly associated with changes in the protonation state and net charge of the adsorbent surface. Below the point of zero charge, the surface is generally more positively charged and therefore more favorable for anion adsorption. In contrast, above the point of zero charge, the surface becomes increasingly negative [32]. The gradual increase in removal from pH 2 to pH 5 indicates that maximum bromide uptake did not occur under the most strongly acidic conditions. Excessive acidity may alter the accessibility or chemical condition of some adsorption sites. In addition, bromide adsorption by a composite material may involve more than electrostatic attraction, including pore adsorption and interactions with alumina-, silica-, or calcium-containing surface sites. Alumina-based materials have been reported to effectively remove bromide, and their performance was strongly influenced by solution pH, supporting the contribution of mineral surface sites to bromide adsorption [28]. The sharp reduction in Br^−^ removal above pH 5 can be explained by progressive deprotonation of the CAKS surface: ≡SOH ⇌ ≡SO^−^ + H^+^.

As the pH increased, the number of positively charged sites decreased, while neutral and negatively charged sites became more abundant. Consequently, the electrostatic attraction between the adsorbent and Br^−^ weakened. At alkaline pH, the negatively charged surface may repel Br^−^ ions, and OH^−^ ions may also compete with Br^−^ for available adsorption sites. This explains the substantial decrease in removal efficiency from approximately 97.34% at pH 5 to approximately 36.07% at pH 10. Similar pH-dependent behavior has been observed for adsorption on mineral oxide surfaces, where increasing pH and reversing the surface charge reduced the adsorption of anionic species [31].

The point of zero charge was estimated from the plot of ΔpH against initial pH. The point of zero charge corresponds to the initial pH at which ΔpH = 0. In the present study, ΔpH was slightly positive at pH 5 and became negative at pH 6. The curve, therefore, crossed the zero line between these two values. Based on linear interpolation, the apparent point of zero charge of the CAKS composite was approximately 5.5. This value indicates that the CAKS surface was predominantly positively charged below pH 5.5 and predominantly negatively charged above pH 5.5. It should be described as an apparent point of zero charge, because the terminology and experimental method used to determine PZC must be interpreted carefully [32].

The point-of-zero-charge result agrees well with the bromide adsorption data. Maximum Br^−^ removal occurred at pH 5, which is slightly below the estimated pH_PZC_ of 5.5. At this pH, the CAKS surface remained slightly positively charged and was therefore favorable for attracting Br^−^ ions. When the solution pH exceeded the pH_PZC,_ the surface became negatively charged, leading to weaker electrostatic attraction and greater repulsion of Br^−^. The close agreement between the maximum adsorption pH and the measured surface-charge transition supports a possible contribution of electrostatic interactions to the adsorption process.

Activated carbon has previously been used to remove bromide from water, although its efficiency strongly depends on surface chemistry, pore structure, water composition, and adsorbent modification. Reference [33] reported that granular activated carbon reduced bromide concentrations in both bench- and pilot-scale experiments, confirming that carbonaceous adsorbents can contribute to bromide removal from aqueous systems. The presence of kaolin and calcium-containing phases in the CAKS composite may provide additional adsorption sites beyond those available on conventional activated carbon.

#### 3.3.2. Effect of Adsorbent Dosage

**Figure 7:**
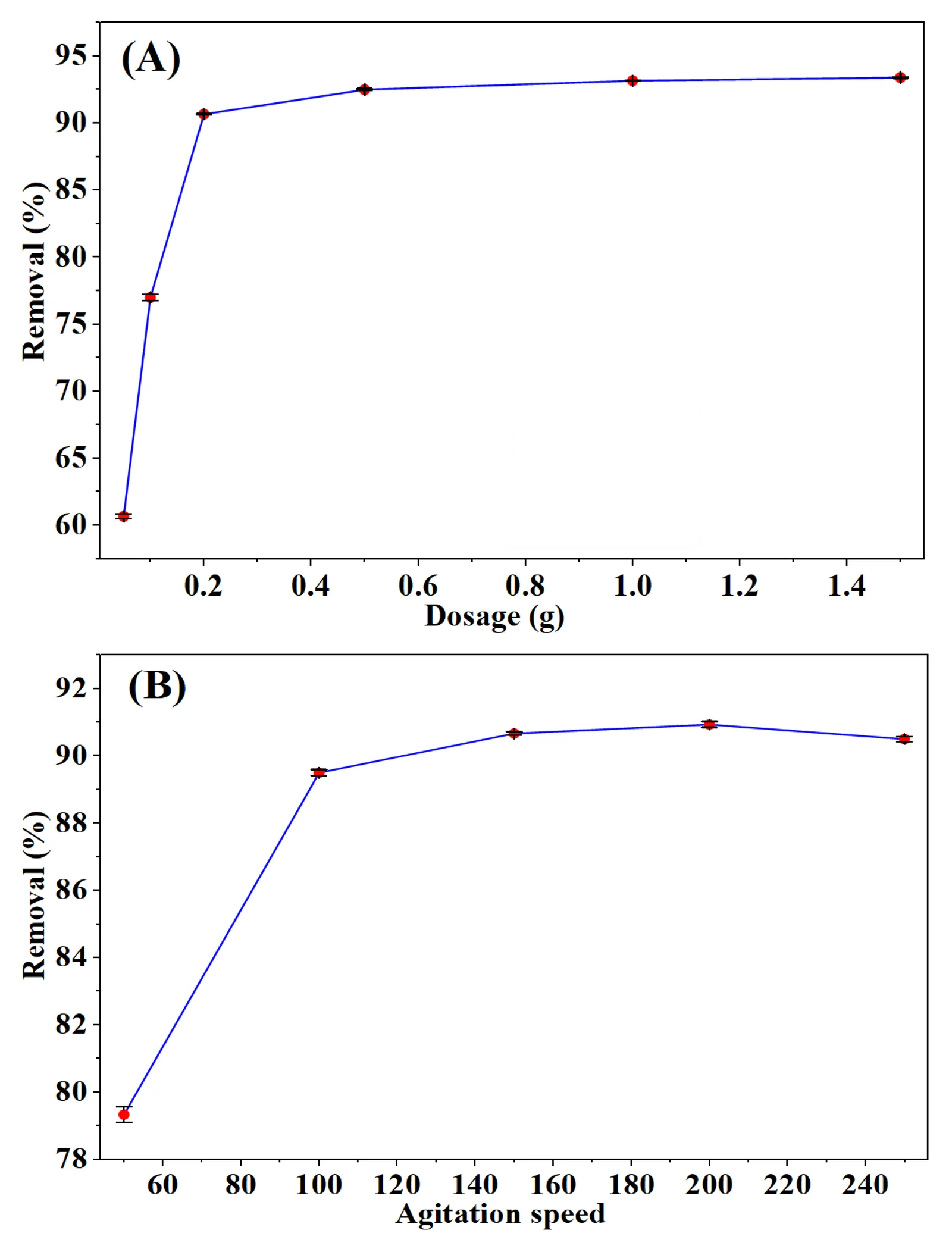

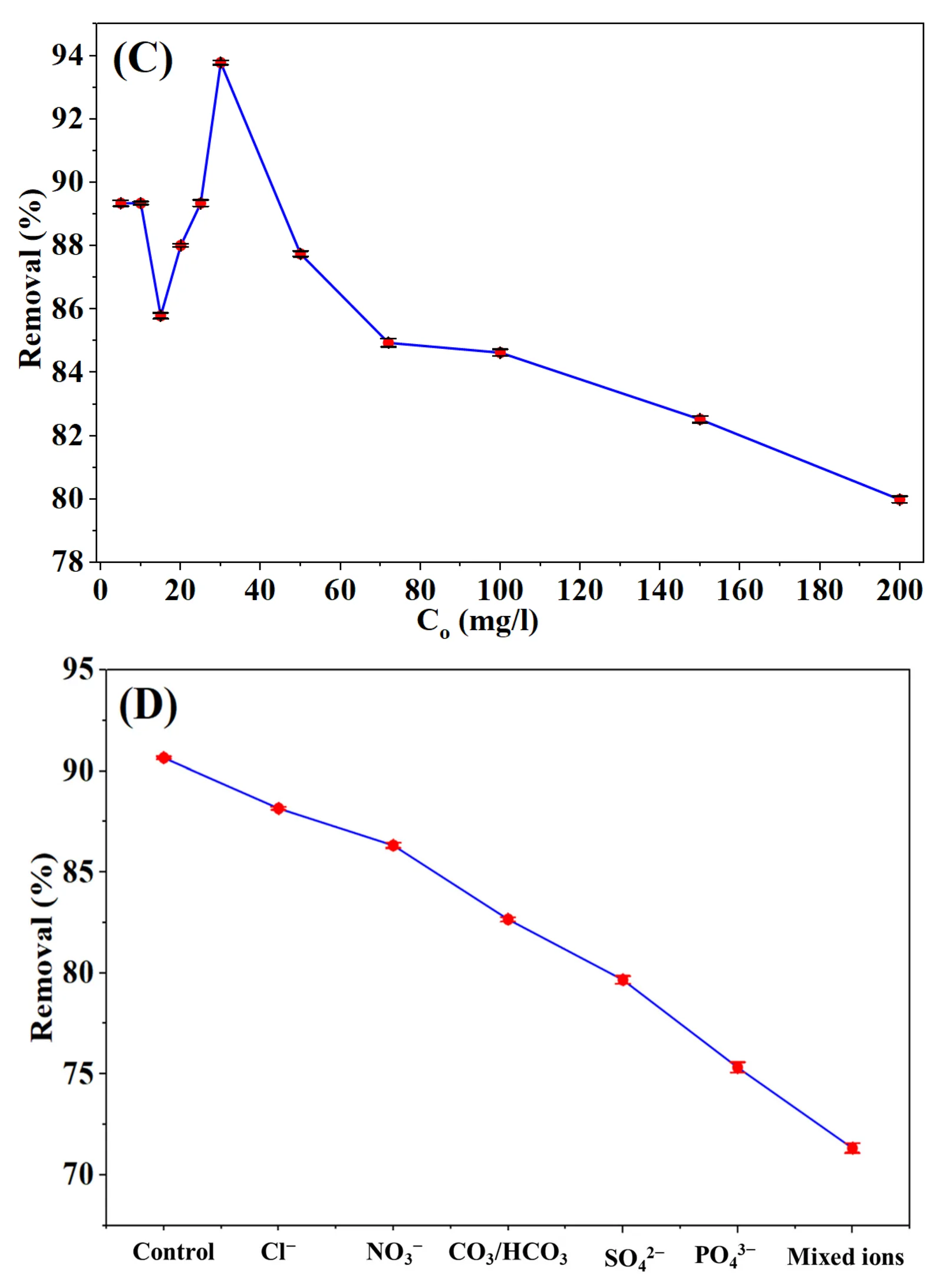
Effect of (**A**) CAKS adsorbent dosage, (**B**) agitation speed, (**C**) initial bromide concentration, and (**D**) coexisting ions on bromide removal efficiency under the selected experimental conditions. Data are presented as mean ± Standard deviation (SD) (n = 3).

The effect of CAKS dosage on bromide removal is shown in Figure 7A. Increasing the adsorbent dosage from 2.0 to 60.0 g L^−1^, corresponding to 0.05–1.50 g of CAKS in 25 mL of solution, increased the bromide removal efficiency from 60.67% to 93.40%. A pronounced increase was observed up to 8.0 g L^−1^ (0.20 g), where the removal efficiency reached 90.67%. Further increases in adsorbent dosage resulted in only marginal improvements, with removal efficiencies of 92.50%, 93.17%, and 93.40% at 20.0, 40.0, and 60.0 g L^−1^, respectively. These results indicate that the adsorption system approached a removal plateau at the higher adsorbent dosages.

In contrast, the equilibrium adsorption capacity decreased continuously from 9.10 mg g^−1^ at 0.05 g to 0.467 mg g^−1^ at 1.50 g. At low dosage, a larger amount of bromide was available per unit mass of adsorbent, resulting in higher site utilization. At higher dosages, the fixed amount of bromide was distributed over a greater mass of adsorbent, leaving many adsorption sites unsaturated. Particle aggregation and overlapping of adsorption surfaces may also reduce the effective surface area at excessive dosage. This inverse relationship between removal efficiency and adsorption capacity is commonly observed in batch adsorption systems using activated carbon and mineral-based adsorbents [1,13].

Although the highest removal efficiency was obtained at 60.0 g L^−1^ (1.50 g/25 mL), a dosage of 20.0 g L^−1^ (0.50 g/25 mL) may be considered a practical operating dosage because it achieved 92.50% bromide removal, only 0.90 percentage points below the maximum value, while requiring only one-third of the adsorbent mass used at 60.0 g L^−1^. Therefore, 20.0 g L^−1^ provides a more favorable balance between bromide removal efficiency and adsorbent consumption.

#### 3.3.3. Effect of Agitation Speed

Increasing the agitation speed from 50 to 200 rpm improved bromide removal from approximately 79.3% to 90.93%, owing to better mixing and reduced external mass-transfer resistance (Figure 7B). Above 150 rpm, the improvement was small, indicating that film diffusion was largely minimized and adsorption became controlled by site availability or intraparticle diffusion. The slight decrease at 250 rpm may be related to excessive turbulence or desorption of weakly bound bromide. Therefore, 200 rpm was selected as the optimum speed, although 150 rpm provided nearly comparable removal with lower energy demand. Similar agitation effects have been reported for bromide adsorption systems, where increasing mixing enhanced mass transfer until a limiting speed was reached [34,35].

#### 3.3.4. Effect of Initial Concentration

The effect of initial bromide concentration on removal by the CAKS composite was investigated over the range of 5–200 mg L^−1^. As shown in Figure 7C, the removal efficiency exhibited moderate variability at the lower initial concentrations. Removal efficiencies of approximately 89.34%, 89.34%, 85.79%, 88.01%, and 89.34% were obtained at initial bromide concentrations of 5, 10, 15, 20, and 25 mg L^−1^, respectively. The highest removal efficiency, approximately 93.78%, was observed at an initial concentration of 30 mg L^−1^. Above 30 mg L^−1^, the removal efficiency progressively decreased to approximately 87.75%, 84.94%, 84.63%, 82.53%, and 79.99% at initial bromide concentrations of 50, 72, 100, 150, and 200 mg L^−1^, respectively. Despite some variability at the lower concentrations, a consistent decline in removal efficiency was observed at concentrations above 30 mg L^−1^.

The decrease in removal efficiency at the higher initial bromide concentrations indicates progressive saturation of the readily accessible adsorption sites on the CAKS composite. As the number of bromide ions increased relative to the number of available adsorption sites, a larger fraction of bromide remained in solution at equilibrium. Increased competition among bromide ions for available surface sites and greater diffusion resistance within the internal pore network may also have contributed to this trend. Nevertheless, the adsorption capacity increased with increasing initial bromide concentration because of the greater amount of bromide available per unit mass of adsorbent. The non-monotonic behavior observed at lower concentrations may reflect the heterogeneous distribution of adsorption sites within the composite and experimental variability.

#### 3.3.5. Effect of Coexisting Ions

The presence of coexisting anions decreased bromide adsorption because these species competed with Br^−^ for positively charged hydroxyl groups, calcium-containing sites, and aluminosilicate surface sites on the CAKS composite (Figure 7D and Table S3). Chloride and nitrate produced relatively weak interference because they are monovalent ions and generally form weak outer-sphere electrostatic associations with oxide surfaces. In contrast, carbonate/bicarbonate, sulfate, and phosphate caused stronger inhibition because their higher charge densities promote stronger electrostatic attraction and surface complexation [36,37].

Phosphate is expected to exhibit particularly strong competition because it can form inner-sphere complexes with aluminum hydroxyl groups and interact with calcium-containing components; however, formation of specific calcium–phosphate species was not directly verified. Carbonate may also interact with calcium-associated surface domains and alter the surface charge via pH changes, while sulfate can strongly occupy protonated hydroxyl sites. Consequently, these multivalent ions reduce the number of active sites available for bromide adsorption. The mixed-ion solution caused the greatest reduction because simultaneous competition and increased ionic strength weakened the electrostatic attraction between Br^−^ and the CAKS surface. Similar effects of competing anions have been reported for bromide adsorption on alumina and layered double hydroxide materials [28,38].

### 3.4. Kinetic Behavior

**Figure 8:**
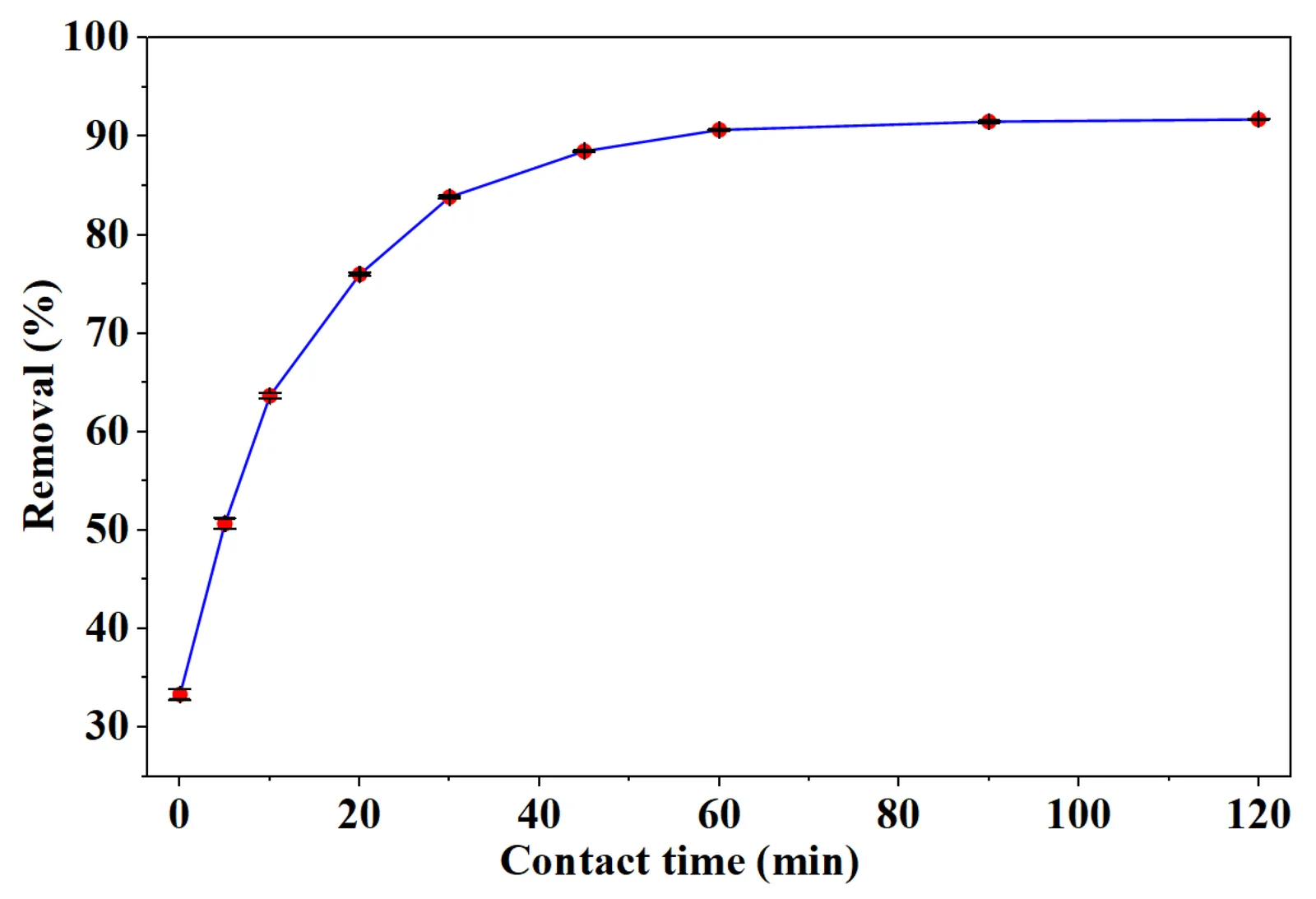
Effect of contact time on bromide removal efficiency by the CAKS composite under the selected experimental conditions. Data are presented as mean ± Standard deviation (SD) (n = 3).

The contact time curve shows that bromide removal by the CAKS composite increased rapidly during the initial stage of adsorption and then gradually approached equilibrium (Figure 8 and Table S4).

At the beginning of the experiment, the removal efficiency was approximately 33%. It increased sharply to about 50% after 5 min, 64% after 10 min, and nearly 76% after 20 min. This rapid initial uptake can be attributed to the large number of vacant active sites on the adsorbent’s external surface. The removal efficiency subsequently increased to approximately 83.83% after 30 min and 88.50% after 45 min. After 60 min, the removal efficiency reached approximately 90.67%. Only a small additional increase occurred thereafter, reaching approximately 91.50% at 90 min and 91.73% at 120 min, indicating that the adsorption system had essentially approached equilibrium. The slower adsorption rate during the later stage may be attributed to the reduced availability of vacant adsorption sites and the longer diffusion pathways required for bromide ions to access internal pores.

The adsorption kinetics of bromide ions onto the CAKS composite were investigated at initial Br^−^ concentrations ranging from 10 to 50 mg L^−1^ (Figure S2 and Table 2). The adsorption process was initially rapid, then gradually slowed until equilibrium was reached. This behavior can be attributed to the large number of available positively charged and calcium-containing active sites at the beginning of the experiment. As these sites became progressively occupied, the remaining Br^−^ ions had to diffuse into the composite’s internal pores, thereby slowing the adsorption rate.

The experimental equilibrium adsorption capacity, q_e_, exp, increased from 2.3667 to 11.3550 mg g^−1^ as the initial Br^−^ concentration increased from 10 to 50 mg L^−1^. This increase was due to a higher concentration gradient between the aqueous phase and the adsorbent surface, which enhanced the mass-transfer driving force and promoted the migration of Br^−^ ions toward the CAKS active sites.

The pseudo-first-order kinetic model showed relatively low coefficients of determination, with R^2^ values ranging from 0.8214 to 0.9394. Moreover, the calculated adsorption capacities were significantly lower than the experimental values. For instance, at an initial concentration of 50 mg L^−1^, the calculated capacity was 6.7248 mg g^−1^, whereas the experimental value was 11.3550 mg g^−1^. Therefore, this model was unable to adequately describe the overall Br^−^ adsorption process.

In contrast, the pseudo-second-order model exhibited excellent agreement with the experimental data. The R^2^ values ranged from 0.9978 to 0.9992, while the calculated equilibrium capacities varied from 2.4242 to 11.5875 mg g^−1^ and were very close to the corresponding experimental values. The pseudo-second-order rate constant, K_2_, decreased from 0.2806 to 0.0474 g mg^−1^ min^−1^ with increasing initial Br^−^ concentration. This decrease suggests that the relative number of available active sites became smaller at higher bromide concentrations. The good model fit indicates that the adsorption rate was strongly dependent on the availability of surface sites and Br^−^–surface interactions. However, it should not be considered definitive evidence of pure chemisorption. The intraparticle-diffusion constant, K_p_, increased from 0.1261 to 0.5773 mg g^−1^ min^−1^/^2^ as the initial concentration increased. This trend confirms that a stronger concentration gradient enhances Br^−^ diffusion into the porous structure.

Nevertheless, the intraparticle-diffusion plots did not pass through the origin, indicating that pore diffusion was not the only rate-controlling step. The film-diffusion model also yielded relatively high R^2^ values ranging from 0.8962 to 0.9928. This result demonstrates that Br^−^ transport through the liquid boundary layer surrounding the CAKS particles contributed to the adsorption process. The bromide uptake occurred through a multistep mechanism involving external mass transfer, electrostatic attraction to protonated surface groups, interaction with calcium-rich sites, and intraparticle diffusion through the porous composite structure.

### 3.5. Isotherm Behavior

**Figure 9:**
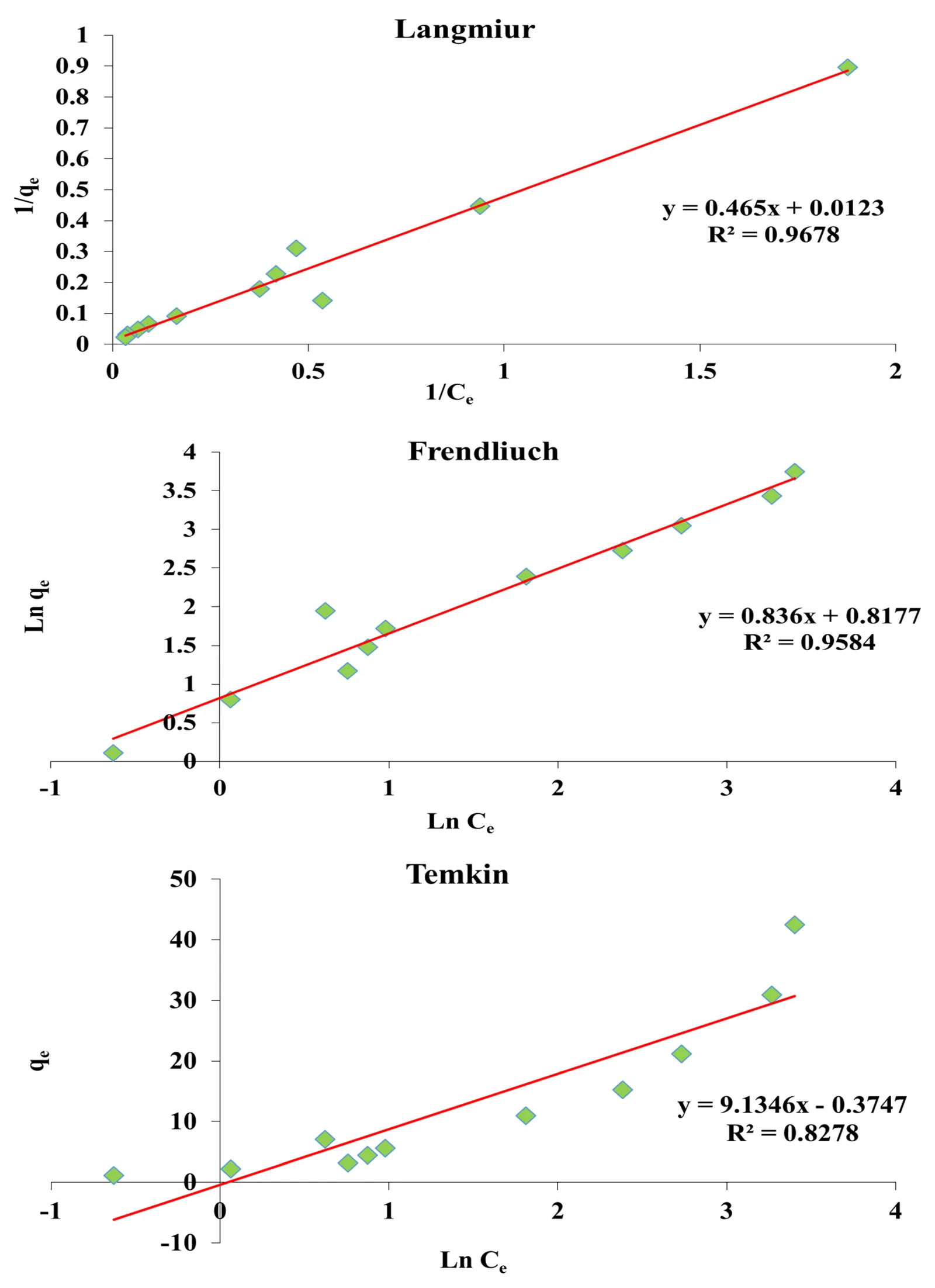

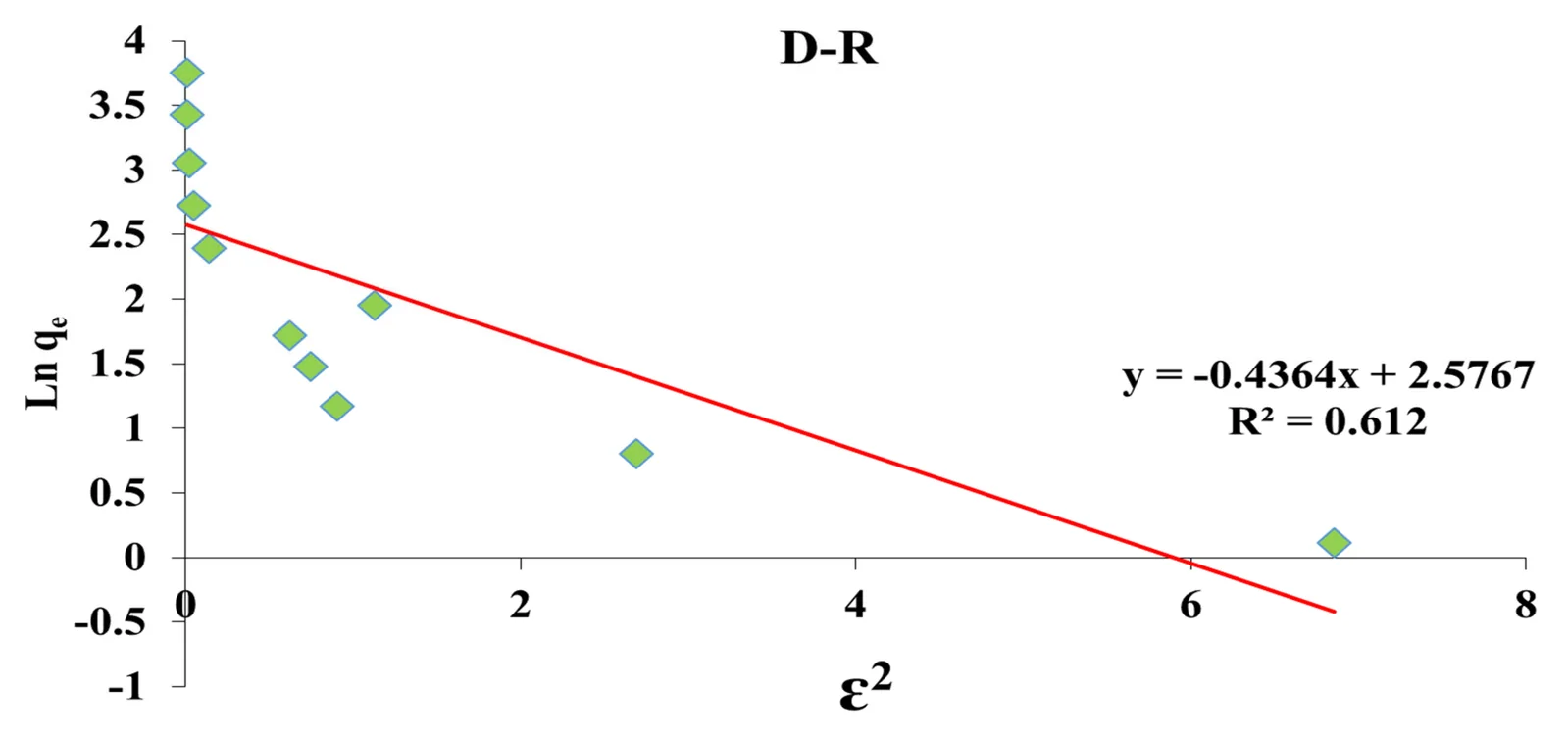
Linearized adsorption-isotherm plots for bromide uptake by the CAKS composite at 298 K: Langmuir, Freundlich, Temkin, and Dubinin–Radushkevich models. The green diamond symbols represent the experimental data points, while the red solid line represents the linear regression fit.

The equilibrium adsorption data for bromide uptake by the CAKS composite were analyzed using the Langmuir, Freundlich, Temkin, and Dubinin–Radushkevich (D–R) isotherm models at 298 K (Figure 9 and Table 3). The expanded concentration range resulted in a maximum experimentally observed adsorption capacity of approximately 40.00 mg g^−1^ at an initial bromide concentration of 200 mg L^−1^. The calculated isotherm parameters and statistical indicators are summarized in Table 3.

The Langmuir model provided a good description of the equilibrium data and exhibited the highest coefficient of determination among the investigated models (R^2^ = 0.9678). The model yielded a maximum monolayer adsorption capacity, q_max_ of 81.30 mg g^−1^ and a Langmuir affinity constant, K_L_ of 0.0264 L mg^−1^. The corresponding RMSE, AARE, and χ^2^ values were 2.7107 mg g^−1^, 13.4003%, and 5.2702, respectively. The model-estimated q_max_ was approximately 2.03 times the maximum experimentally observed adsorption capacity of 40.00 mg g^−1^. Therefore, the Langmuir q_max_ should be considered a model-derived extrapolated capacity rather than a directly measured adsorption capacity [39].

The Freundlich model also described the equilibrium data satisfactorily and showed a fit comparable to that of the Langmuir model. The Freundlich constants were K_F_ = 2.2652, 1/n = 0.8360, and nn = 1.1961. The value of 1/n between zero and one indicates favorable adsorption, while its deviation from unity is consistent with adsorption on a heterogeneous surface containing sites with different adsorption energies. The Freundlich model yielded R^2^ = 0.9584, RMSE = 2.3855 mg g^−1^, AARE = 14.6193%, and χ^2^ = 4.6093.

Comparison of the statistical indicators shows that neither the Langmuir nor the Freundlich model was superior according to every error criterion. The Langmuir model exhibited the higher R^2^ value and the lower AARE, whereas the Freundlich model produced lower RMSE and χ^2^ values. Thus, both models provided reasonably close descriptions of the equilibrium data. The slightly higher R^2^ of the Langmuir model suggests a predominantly finite-capacity adsorption behavior, whereas the comparable Freundlich fit supports the heterogeneous nature of the CAKS surface.

The Temkin model yielded A_T_ = 1.0418 L g^−1^, B_T_ = 9.1346 mg g^−1^, and R^2^ = 0.8278. The corresponding RMSE and AARE values were 5.3132 mg g^−1^ and 94.3989%, respectively. The Temkin equation generated a negative predicted adsorption capacity at the lowest equilibrium concentration; therefore, the corresponding χ^2^ value was not considered valid because the predicted value occurs in the denominator of the χ^2^ expression. The relatively lower R^2^ and larger error values indicate that the Temkin model was less suitable for describing bromide adsorption over the investigated concentration range.

The Dubinin–Radushkevich model produced a theoretical saturation capacity, qmq_m, of 13.1537 mg g^−1^ and a D–R constant, β, of 0.4364 mol^2^ kJ^−2^. The model showed the weakest overall agreement with the experimental data, with R^2^ = 0.6120, RMSE = 11.0660 mg g^−1^, AARE = 63.3017%, and χ^2^ = 106.81. In addition, the estimated D–R saturation capacity was substantially lower than the experimentally measured adsorption capacities at the higher initial bromide concentrations. Therefore, the D–R model did not adequately represent bromide adsorption over the investigated concentration range [39,40].

Overall, the Langmuir and Freundlich models provided substantially better descriptions of the equilibrium data than the Temkin and D–R models. The results suggest that bromide uptake can be represented as adsorption toward a finite apparent capacity while occurring on a chemically and structurally heterogeneous composite surface. Accordingly, the equilibrium behavior is consistent with adsorption on carbonaceous, aluminosilicate, and calcium-associated surface domains having different adsorption environments. The Langmuir-derived maximum capacity should nevertheless be interpreted cautiously because the predicted q_max_ of 81.30 mg g^−1^ exceeds the maximum experimentally observed value of approximately 40.00 mg g^−1^ [28,38].

### 3.6. Thermal Behavior

**Figure 10:**
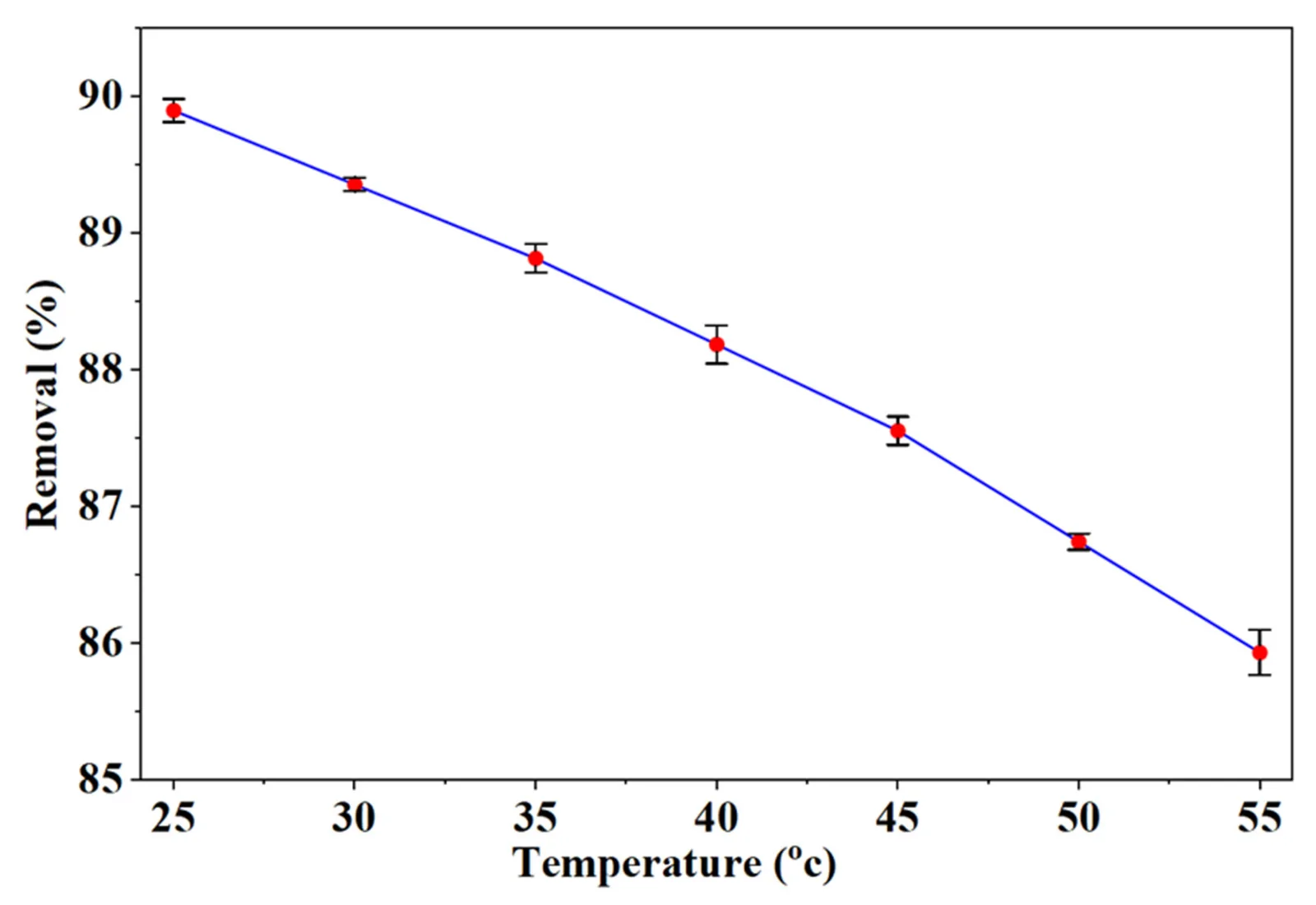
Effect of temperature on bromide removal by the CAKS composite and the Van’t Hoff plot used to determine the apparent thermodynamic parameters.

The influence of temperature on bromide adsorption by the CAKS composite was investigated over the range of 25–55 °C (Figure 10 and Table S4). A gradual decline in adsorption performance was observed as the temperature increased. The equilibrium adsorption capacity decreased from 6.743 mg g^−1^ at 25 °C to 6.445 mg g^−1^ at 55 °C, while the removal efficiency decreased from 89.90% to 85.93% over the same temperature range. This trend indicates that higher temperatures were unfavorable for bromide uptake, most likely because increased thermal motion weakened interactions between bromide ions and the composite’s active sites and promoted the release of weakly retained ions back into solution.

The Van’t Hoff plot exhibited a strong linear correlation, with an R^2^ value of 0.9948, indicating that the temperature dependence of bromide adsorption was satisfactorily described within the investigated range (Figure 10 and Table 4). The calculated ΔH_app_ = −10.15 ± 0.234 kJ mol^−1^ and an apparent entropy change of ΔS_app_ = −15.78 ± 0.750 J mol^−1^ K^−1^, respectively. The negative ΔH_app_ value is consistent with an apparent exothermic adsorption behavior over the investigated temperature range.

The apparent enthalpy change was negative, which is consistent with an apparent exothermic temperature dependence of bromide uptake under the investigated conditions. The relatively small magnitude of is compatible with comparatively weak overall adsorbate–surface interactions. However, because this parameter was derived from an operational concentration-based (K_c_), its magnitude should not be used independently to distinguish physisorption from chemisorption or to assign a specific adsorption mechanism. Thus, contributions from electrostatic interactions, surface association, pore-related uptake, and other interactions cannot be differentiated solely from the thermodynamic data.

The negative ΔS_app_ value indicates a decrease in randomness at the solid–solution interface during bromide adsorption. The negative ΔS_app_ value suggests an apparent decrease in interfacial disorder during Br^−^ adsorption under the investigated conditions. Such behavior may be attributed to the transfer of freely mobile bromide ions from the aqueous phase to more organized surface-associated states on the CAKS composite.

The Gibbs free-energy values were negative throughout the investigated temperature range, decreasing in magnitude from −5.42 kJ mol^−1^ at 25 °C to −4.95 kJ mol^−1^ at 55 °C. The negative ΔG_app_ values indicate that Br^−^ adsorption onto CAKS was thermodynamically favorable under the investigated experimental conditions. However, the progressive shift toward less negative values as temperature increases indicates that the thermodynamic driving force for adsorption weakens at higher temperatures.

The apparent thermodynamic parameters indicate that bromide uptake was thermodynamically favorable within the investigated experimental conditions and exhibited an apparent exothermic temperature dependence. The decreasing adsorption performance with increasing temperature is consistent with more favorable uptake at lower temperatures. However, because the thermodynamic parameters were derived from an operational concentration-based equilibrium ratio, they should not be interpreted as definitive evidence for a particular adsorption mechanism. The relatively small magnitude of ΔH_app_ is consistent with relatively weak overall interactions but does not, by itself, distinguish physisorption from chemisorption or exclude contributions from other surface interactions. When considered together with the pH-dependent adsorption behavior and apparent pH_PZC_, electrostatic interactions may contribute to bromide uptake, although the available data do not establish them as the exclusive or dominant mechanism.

### 3.7. Regeneration and Reusability

**Figure 11:**
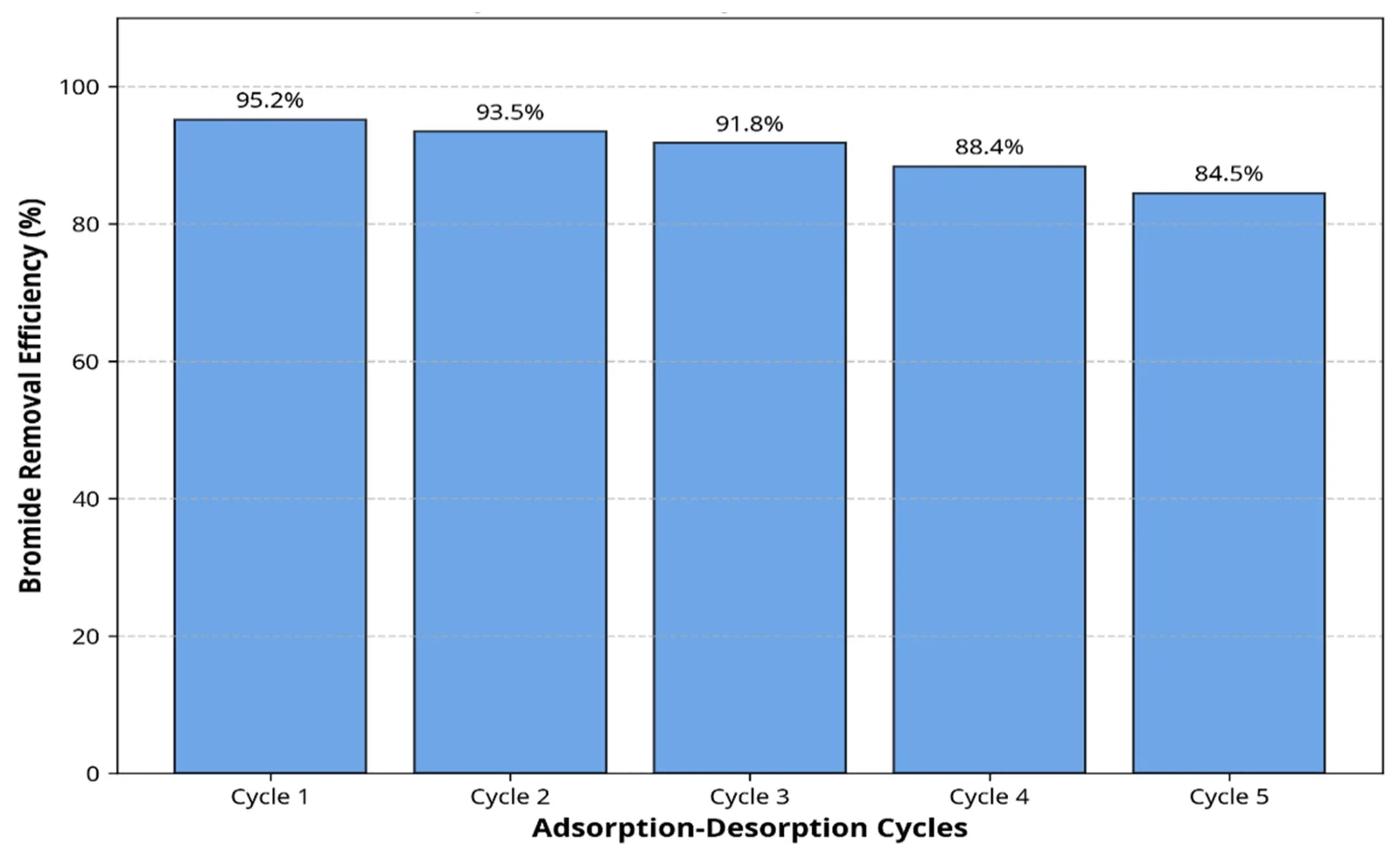
Bromide removal efficiency of the CAKS composite over five consecutive adsorption–desorption cycles.

The regeneration performance of the CAKS composite was evaluated over five consecutive adsorption–desorption cycles to assess its potential for repeated use in treating bromide-contaminated water. All adsorption–desorption cycles were conducted under identical adsorption conditions to ensure direct comparison of the regeneration performance. After each adsorption cycle, the bromide-loaded composite was treated with 0.1 M NaOH, washed thoroughly with deionized water, and heated at 60 °C before reuse. The change in bromide removal efficiency over the five cycles is shown in Figure 11. The CAKS composite maintained high bromide removal during the first three cycles. The removal efficiency decreased only slightly from 95.2% in the first cycle to 91.8% in the third cycle. After five cycles, the composite still achieved approximately 84.5% bromide removal. This corresponds to a reduction of 10.7 percentage points, or about 11.2% relative to the initial performance. The moderate decline indicates that most adsorption sites remained active and accessible after repeated regeneration.

The effectiveness of NaOH regeneration is primarily related to changes in the composite’s surface charge. Under mildly acidic adsorption conditions, many surface hydroxyl groups become positively charged and attract bromide ions through electrostatic interactions and outer-sphere ion association. During regeneration, the alkaline solution removes protons from these surface groups, reducing the positive charge and weakening the interaction between Br^−^ and the CAKS surface. As a result, a substantial fraction of the adsorbed bromide is released into the regenerate solution. Similar behavior has been reported for bromide-removing ion-exchange and mineral-based adsorbents regenerated using alkaline or saline solutions [34,38].

Several factors may cause the gradual reduction in performance over repeated cycles. Some bromide ions may remain trapped in narrow or poorly connected pores and may not be fully removed during regeneration. Bromide associated with less accessible aluminosilicate or calcium-containing sites may also require longer regeneration time or a stronger desorbing solution. Repeated exposure to alkaline conditions may slightly modify surface hydroxyl groups or promote limited leaching of weakly bound calcium species. In addition, residual sodium ions or other dissolved species may remain within the porous structure and occupy part of the available adsorption sites during the following cycle.

### 3.8. Comparison of Bromide Adsorbents

The development of efficient adsorbents for bromide removal is important for reducing the formation of brominated disinfection byproducts during water treatment. Table 5 compares the reported adsorption capacities of CAKS with those of previously reported bromide adsorbents, along with the corresponding experimental conditions, including initial bromide concentration, pH, adsorbent dosage, temperature, and contact time.

The CAKS composite exhibited a Langmuir model-estimated maximum adsorption capacity (qmax) of 81.30 mg g^−1^. However, the adsorption capacities reported in the literature were obtained under different experimental conditions, which can substantially influence adsorption performance. Therefore, the values presented in Table 5 are intended to provide contextual information rather than a direct ranking of adsorbent performance. Direct comparison or claims of superiority based solely on qmax should be avoided unless the adsorbents are evaluated under equivalent experimental conditions.

Nevertheless, CAKS exhibited appreciable bromide adsorption and was prepared from readily available components, including kaolin, activated carbon, a calcium precursor, and starch. Its performance should therefore be considered alongside material availability, porous structure, preparation simplicity, and reusability, rather than adsorption capacity alone.

### 3.9. Proposed Mechanisms of Bromide Sequestration

**Figure 12:**
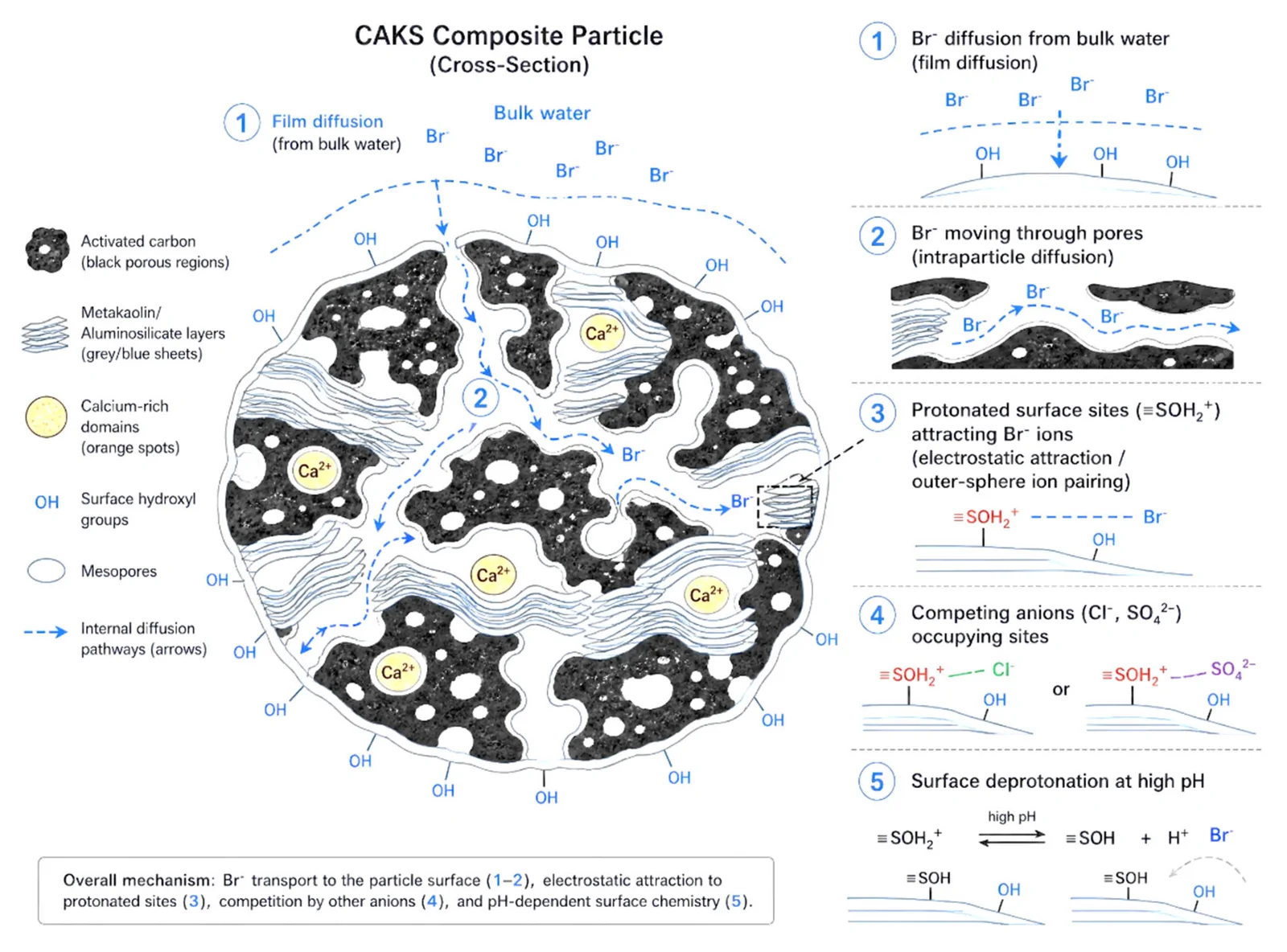
Proposed mechanism of bromide adsorption onto the CAKS composite. Bromide ions are transported from the bulk solution to the external particle surface by film diffusion and subsequently migrate through interconnected mesopores by intraparticle diffusion. Under mildly acidic conditions, protonated hydroxyl sites on activated carbon, metakaolin/aluminosilicate phases, and calcium-rich domains attract Br^−^ through electrostatic interactions and outer-sphere ion pairing. Bromide retention is further supported by pore filling and weak association with hydrated surface sites. Competing anions occupy the same adsorption sites, while surface deprotonation above the point of zero charge weakens bromide uptake through electrostatic repulsion.

The removal of bromide via the CAKS composite is governed by a complex interplay of physical and chemical interactions, facilitated by its heterogeneous surface chemistry (Figure 12). The experimental results are consistent with a multistep bromide uptake process involving electrostatic interactions, mass transfer, pore-associated adsorption, and possible interactions with mineral and calcium-containing surface domains. However, these pathways should be regarded as proposed mechanisms because direct molecular-level evidence for surface complexation, ion exchange, or specific bromine-containing surface species was not obtained in the present study.

The CAKS framework contains kaolin-derived aluminosilicate domains and detectable calcium; however, the exact calcium-containing phase was not conclusively identified. At pH levels below the point of zero charge, the surface functional groups, particularly the aluminol (Al-OH) and silanol (Si-OH) groups on the kaolin and, potentially, on hydroxylated calcium-associated surface sites, become protonated, carrying a net positive charge. This creates a strong electrostatic gradient that facilitates the migration of the anionic Br^−^ from the bulk solution to the adsorbent surface. Unlike larger oxyanions, the relatively small ionic radius of bromide allows it to approach these active sites closely, where it may participate in weak outer-sphere association.

Calcium was detected in the composite, but its precise contribution to bromide uptake cannot be isolated from the present data. Calcium-containing surface domains may participate in weak electrostatic or outer-sphere interactions; however, formation of calcium–bromide complexes, hydrated-lime species, or lattice incorporation was not directly demonstrated.

The architectural role of starch and activated carbon ensures that these chemical sites are accessible. The decomposition of starch during calcination creates a hierarchical macroporous structure that reduces the path length for intraparticle diffusion. This allows bromide ions to penetrate deep into the composite interior, engaging with internal active sites that would otherwise be shielded. Consequently, the mechanism is a hybrid process where the carbonaceous framework provides the structural “scaffold” and high surface area. In contrast, the mineral and calcium components provide the “chemical traps” necessary for effective bromide immobilization.

### 3.10. Limitations and Future Research

Several limitations of the present study should be considered when interpreting the performance and proposed adsorption mechanisms of CAKS. First, although calcium was detected in the composite, the specific chemical phase and coordination environment of the calcium-containing domains were not conclusively identified; therefore, their precise contribution to bromide uptake remains uncertain. Second, the adsorption experiments were conducted predominantly using synthetic bromide solutions prepared in deionized water. Although selected competing inorganic ions were evaluated, these experiments do not fully represent the complexity of natural waters, where natural organic matter, variable ionic strength, and multiple coexisting species may influence bromide uptake. Third, the proposed contributions of electrostatic attraction, surface association, ion exchange, and interactions with calcium- and aluminosilicate-containing domains are inferred from adsorption behavior, pH-dependent trends, kinetic analysis, FTIR, and elemental characterization rather than directly confirmed at the molecular level. Accordingly, future studies should evaluate CAKS using representative natural water matrices and apply high-resolution XPS, detailed elemental mapping, solid-state NMR where applicable, and bromine chemical-state/speciation analysis to clarify the bromide-binding environment and distinguish among possible uptake mechanisms.

Component-specific adsorption experiments were not conducted in the present study; therefore, the individual contributions of the kaolin-derived, carbonaceous, calcium-containing, and starch-derived structural components to bromide uptake cannot be quantitatively distinguished. Future studies should evaluate the individual constituents and appropriate control materials under identical adsorption conditions to determine their relative contributions and possible synergistic effects, thereby providing further validation of the proposed bromide-sequestration mechanism.

## 4. Conclusions

This study developed a low-cost hybrid composite, denoted CAKS, from kaolin, activated carbon, a calcium precursor, and starch for the removal of bromide from aqueous media. The starch-assisted consolidation and calcination process produced a porous, heterogeneous material containing mineral, carbonaceous, and calcium-rich domains. Characterization results showed that CAKS possessed a mesoporous structure with a BET surface area of 212.5 m^2^ g^−1^, a total pore volume of 0.384 cm^3^ g^−1^, and an average pore diameter of 7.2 nm. These properties provided accessible pathways and multiple surface sites for bromide uptake.

Batch experiments demonstrated that bromide removal was strongly influenced by solution pH, adsorbent dosage, contact time, initial concentration, agitation speed, temperature, and the presence of competing ions. The highest removal efficiency, approximately 97.3%, was achieved at pH 5, which was close to the apparent point of zero charge of 5.5.

This agreement suggests that electrostatic attraction between bromide ions and positively charged surface sites may contribute to the adsorption process, although its relative contribution cannot be quantified from the available data. Bromide uptake was rapid during the first stage of contact and approached equilibrium within 60–90 min. The maximum experimentally measured adsorption capacity was approximately 40.0 mg g^−1^ at an initial bromide concentration of 200 mg L^−1^, whereas the Langmuir model yielded an extrapolated q_max_ of 81.30 mg g^−1^.

The pseudo-second-order model best described the kinetic results; however, the contributions of film and intraparticle diffusion indicate that bromide uptake occurred through a multistep process rather than a single rate-controlling mechanism. The equilibrium behavior was consistent with adsorption on a chemically heterogeneous surface. Although the Langmuir model yielded a high theoretical capacity, this value substantially exceeded the measured range and should therefore be interpreted cautiously. The experimentally observed capacity provides a more reliable indication of current performance.

Compared with previously reported bromide adsorbents, CAKS provides a favorable balance between adsorption performance, precursor availability, structural characteristics, and reusability. However, the adsorption capacities reported in the literature were obtained under different experimental conditions, including variations in initial bromide concentration, pH, adsorbent dosage, temperature, and contact time. Therefore, direct ranking of CAKS against other adsorbents based solely on qmax is not appropriate. The performance of CAKS should instead be considered alongside its precursor availability, porous structure, preparation method, rapid bromide uptake, and reusability.

Regeneration with 0.1 M NaOH enabled the composite to be reused over five adsorption–desorption cycles, supporting its potential for repeated application. Nevertheless, further validation is required using natural waters, broader concentration ranges, fixed-bed systems, leaching tests, and direct confirmation of surface-bound bromine. Such studies should also quantify treatment costs and reductions in disinfection byproducts. Overall, CAKS represents a promising low-cost adsorbent for controlling bromide before disinfection, though additional scale-up and mechanistic studies are needed before practical implementation.

## Figures and Tables

**Table 1:** Linearized equations and corresponding parameters of the kinetic, adsorption-isotherm, and thermodynamic models used to evaluate bromide adsorption onto the CAKS composite.

Models	Equations	Parameter Definitions
**1. Kinetic models**		
Pseudo-first-order	ln(q_e_ − q_t_) = ln q_e_ − k_1_t	q_t_ = amount of Br^−^ adsorbed at time t (mg g^−1^) q_e_ = amount of Br^−^ adsorbed at equilibrium (mg g^−1^) k_1_ = pseudo-first-order rate constant (min^−1^) t = contact time (min)
Pseudo-second-order	t/q_t_ = 1/(k_2_q_e_^2^) + t/q_e_	k_2_ = pseudo-second-order rate constant (g mg^−1^ min^−1^) q_t_, q_e_ = adsorbed amount at time t and at equilibrium (mg g^−1^) t = contact time (min)
Intraparticle diffusion	q_t_ = k_p_t^1/2^ + C	k_p_ = intraparticle diffusion rate constant (mg g^−1^ min^−1/2^) C = intercept, related to boundary-layer thickness (mg g^−1^)
Film diffusion	ln(1 − F) = −k_f_t F = q_t_/q_e_	F = fractional attainment of equilibrium at time t k_f_ = film-diffusion rate constant (min^−1^)
**2. Isotherm models**		
Langmuir	1/q_e_ = 1/q_max_ + [1/(q_max_ K_L_)](1/C_e_)	C_e_ = equilibrium Br^−^ concentration in solution (mg L^−1^) q_e_ = equilibrium adsorption capacity (mg g^−1^) q_max_ = maximum monolayer adsorption capacity (mg g^−1^) K_L_ = Langmuir affinity constant (L mg^−1^)
Freundlich	ln q_e_ = ln K_f_ + (1/n) ln C_e_	K_f_ = Freundlich constant, relative adsorption capacity (mg g^−1^)(L mg^−1^)^1/n^ n = Freundlich heterogeneity/intensity parameter (dimensionless)
Temkin	q_e_ = B_T_ ln A_T_ + B_T_ ln C_e_	A_T_ = Temkin equilibrium binding constant (L g^−1^) B_T_ = Temkin constant related to heat of adsorption, B_T_ = RT/b_T_ (mg g^−1^)
Dubinin–Radushkevich	ln q_e_ = ln q_m_ − βε^2^ ε = RT ln (1 + 1/C_e_) E = 1/(2β)^(0.5)^	q_m_ = theoretical saturation capacity (mg g^−1^) β = D–R activity coefficient, related to sorption energy (mol^2^ kJ^−2^) ε = Polanyi adsorption potential E = mean free energy of adsorption per molecule (kJ mol^−1^) R = gas constant (8.314 J mol^−1^ K^−1^); T = absolute temperature (K)
**3. Thermodynamic analysis**		
Distribution coefficient	K_c_ = (C_o_ − C_e_)/C_e_ = (q_e_ ×m)/(C_e_ ×V)	K_c_ = dimensionless concentration-based equilibrium ratio
Gibbs free energy	ΔG_app_ = −RT ln K_c_	ΔG_app_ = apparent Gibbs free-energy change (kJ mol^−1^) R = gas constant (8.314 J mol^−1^ K^−1^); T = absolute temperature (K)
Van’t Hoff	ln K_c_ = ΔS_app_/R − ΔH_app_/(RT)	ΔS_app_ = apparent entropy change; ΔH_app_ = apparent enthalpy change
**4. Error functions**		
Root-mean-square error Average absolute relative error Non-linear chi-square		N = number of experimental data points q_e,exp,i_ = experimentally measured adsorption capacity (mg g^−1^) q_e,cal,i_ = adsorption capacity predicted by the model (mg g^−1^)

**Table 2:** Kinetic parameters for bromide adsorption onto the CAKS composite at different initial bromide concentrations using pseudo-first-order, pseudo-second-order, intraparticle-diffusion, and film-diffusion models.

C_0_ (mg/L)	q_e_ (exp) (mg/g)	Pseudo-First-Order Kinetics			Pseudo-Second-Order Kinetics			Intraparticle Diffusion		Film Diffusion	
		K_1_	q_e_ (cal)	R^2^	K_2_	q_e_ (cal)	R^2^	Kp	R^2^	K	R^2^
10	2.3667	0.1387	1.5310	0.8635	0.2806	2.4242	0.9992	0.1261	0.9507	0.0846	0.9928
20	4.7091	0.1272	2.8994	0.8475	0.1341	4.8146	0.9989	0.2471	0.9672	0.0866	0.9895
30	6.9550	0.1000	3.2398	0.9394	0.0850	7.1174	0.9986	0.3663	0.9760	0.1000	0.9393
40	9.1460	0.1055	4.4119	0.8962	0.0652	9.3284	0.9985	0.4625	0.9843	0.1005	0.8962
50	11.3550	0.1116	6.7248	0.8214	0.0474	11.5875	0.9978	0.5773	0.9918	0.0710	0.9873

**Table 3:** Isotherm parameters and error-function values for bromide adsorption onto CAKS at 298 K.

Model	Parameter	Value	R^2^	RMSE	AARE	χ^2^
				(mg g^−1^)	(%)	
Langmuir	q_max_ (mg g^−1^)	81.3	0.9678	2.7107	13.4003	5.2702
	K_L_ (L mg^−1^)	0.0264				
Freundlich	K_F_	2.2652	0.9584	2.3855	14.6193	4.6093
	1/n	0.836				
	n	1.1961				
Temkin	A_T_ (L g^−1^)	1.0418	0.8278	5.3132	94.3989	N/A ^1^
	B_T_ (mg g^−1^)	9.1346				
Dubinin–Radushkevich	q_m_ (mg g^−1^)	13.1537	0.612	11.066	63.3017	106.81
	β (mol^2^ kJ^−2^)	0.4364				
	E (kJ mol^−1^)	0.5728				

^1^ The Temkin χ^2^ value was not calculated because the model generated a negative predicted q_e_ at the lowest equilibrium concentration.

**Table 4:** Effect of temperature on bromide adsorption capacity, removal efficiency, and apparent thermodynamic parameters of the CAKS composite.

Temperature	q_e_	K_c_	C_e_	R	(∆G_app_)	(∆S_app_)	(∆H_app_)
(°C)	(mg g^−1^)			(%)	(kJ mol^−1^)	(J mol^−1^ K^−1^)	(kJ mol^−1^)
25	6.743	8.9016	3.03	89.9	−5.4195	−15.78 ± 0.750	−10.15 ± 0.234
30	6.702	8.3978	3.1923	89.359	−5.3636		
35	6.661	7.9435	3.3546	88.818	−5.3095		
40	6.614	7.4659	3.544	88.187	−5.2341		
45	6.567	7.0361	3.7333	87.556	−5.1609		
50	6.506	6.5438	3.9768	86.744	−5.0472		
55	6.445	6.1092	4.2203	85.932	−4.9376		

Experimental conditions: C_o_ = 30 mg L^−1^, V = 0.025 L, m = 0.100 g, and m/V = 4.00 g L^−1^.

**Table 5:** Comparison of the reported adsorption capacities and experimental conditions for bromide removal using CAKS and previously reported adsorbents.

Adsorbent Material	q_max_ (mg/g)	Experimental Conditions	Reference
CAKS	81.30	Initial Br^−^ concentration: 5–200 mg L^−1^; pH: 5; adsorbent dosage: 4 g L^−1^ (0.1 g/25 mL); temperature: 25 °C (298 K); contact time: 180 min, with equilibrium approached within 60–90 min	Current Study
gamma-Bi_2_O_3_ Nanostructures	147.10	Initial Br^−^ concentration: 50–500 mg L^−1^; pH: 7; adsorbent dosage: 2 g L^−1^; temperature: 25 °C; contact time: 24 h	[41]
Nano-gamma-Al_2_O_3_	90.72	Initial Br^−^ concentration: 10–100 mg L^−1^; pH: approximately 6; adsorbent dosage: 0.05 g/50 mL (1 g L^−1^); temperature: 25 °C; contact time: 24 h	[28]
Ag-Cu-MOF-74	232.90	Initial Br^−^ concentration: varied in simulated saline water; pH: NR; adsorbent dosage: NR; temperature: NR; contact time: NR for the specific qmax determination	[42]
MIEX Resin	25.10	Initial Br^−^ concentration: varied during equilibrium experiments; pH: 4–9 investigated; adsorbent dosage: varied; temperature: 30 °C (303 K for isotherm analysis); contact time: approximately 60 min to equilibrium	[34]
Ni-Al Layered Double Hydroxides	45.30	Initial Br^−^ concentration: varied during batch/isotherm experiments; pH: reported in the original study; adsorbent dosage: reported in the original study; temperature: varied during thermodynamic experiments; contact time: equilibrium conditions reported in the original study	[38]
Silver-loaded Porous Carbon	1.20	Initial Br^−^ concentration: varied during equilibrium experiments; pH: optimum ≈ 5, with favorable adsorption over pH 4–7; adsorbent dosage: NR for the specific qmax determination; temperature: 25 °C; contact time: equilibrium batch conditions reported in the original study	[43]

## Data Availability

All data supporting the findings of this study are available within the paper and its Supplementary Materials.

## Supplementary Materials

The following supporting information can be downloaded at: https://www.mdpi.com/article/10.3390/polym18202586/s1, Table S1. Verified experimental conditions used for all adsorption experiments, Table S2. Proposed FTIR band assignments and changes after Br^−^ adsorption, Table S3. Effect of coexisting ions on adsorption capacity (q_e_) and removal efficiency (%R), Table S4. Effect of operational parameters, including pH, agitation speed, initial concentration (C_0_), adsorbent dosage, temperature, and contact time, on adsorption capacity (q_e_) and removal efficiency (%R), Figure S1. Nitrogen adsorption–desorption isotherm of the CAKS composite at 77 K, showing a Type IV profile with a distinct hysteresis loop, indicative of a predominantly mesoporous structure, Figure S2. Kinetic analysis of bromide adsorption onto the CAKS composite at different initial concentrations: pseudo-first-order model, pseudo-second-order model, intraparticle-diffusion model, and film-diffusion model.

## References

[1] Xue K., Yang C., He Y. (2023). A review of technologies for bromide and iodide removal from water. Environ. Technol. Rev..

[2] Edition F. (2011). Guidelines for drinking-water quality. WHO Chron..

[3] Alomirah H.F., Al-Zenki S.F., Alaswad M.C., Alruwaih N.A., Wu Q., Kannan K. (2020). Elevated concentrations of bromate in drinking water and groundwater from Kuwait and associated exposure and health risks. Environ. Res..

[4] Lu Y., Wu D.-X., Ye B., Du Y., Wu Q.-Y. (2025). Comparative nontargeted analysis and toxicity of brominated disinfection byproducts from chlorination and chloramination of natural organic matter. Environ. Sci. Technol..

[5] Lan S., Huang W., Kuang D., Qin F., Zhai Y., Wang C., Gao X. (2026). Monitoring and health risk assessment of disinfection byproducts in drinking water in Chengdu, China, 2023–2025. Sci. Rep..

[6] Peterson E.S., Stanford B.D., Raseman W., Russell C., Seidel C., Bruce G. (2026). Disinfection byproducts: Health risks, occurrence, control, and implications for regulatory revision. AWWA Water Sci..

[7] Mendy A. (2025). Disinfection byproducts in US drinking water and cancer mortality. Int. J. Environ. Health Res..

[8] Lippolis A. (2025). From Spectroscopy to Genomics: Dissecting and Predicting Agronomic and Quality Traits in *Vicia faba* L.. Doctoral Dissertation.

[9] Egitto J., Latayan J., Pagsuyoin S., Apul O., Agar E. (2022). Towards selective removal of bromide from drinking water resources using electrochemical desalination. Chem. Eng. J. Adv..

[10] Li Y., Wu J., Yong T., Fei Y., Qi J. (2021). Investigation of bromide removal and bromate minimization of membrane capacitive deionization for drinking water treatment. Chemosphere.

[11] Mkilima T. (2025). Nature-based hybrid adsorbents for emerging contaminant removal: Integrating low-cost bioresources in the water–energy–food nexus. Earth Environ. Sustain..

[12] Tewari S., Kaur J., Tandon S., Das D.K. (2026). Recent advances in functionalized nanocarbon adsorbents for toxic pollutant removal: Challenges and future perspectives. Discov. Nano.

[13] Joo J.-H., Kim S.-H., Kim J.H., Kang H.-J., Lee J.H., Jeon H.-J., Jang Y.H., Lee J.-H., Lee S.-Y., Park S.-J. (2025). Recent advances in activated carbon fibers for pollutant removal. Carbon Lett..

[14] Yousef D., Ahmed A.S., Hamed A.M., Abdelmotallieb M., El-Sayed M.E. (2026). Enhanced wastewater purification using biochar, activated carbon, and kaolinite composites: A multi-parameter approach for sustainable agriculture. Sustainability.

[15] Mestri S., Dogan S., Tizaoui C. (2023). Bromate removal from water using ion exchange resin: Batch and fixed bed column performance. Ozone Sci. Eng..

[16] Rahman N.F.A., Rahman H.A., Azhan W.M.S.W., Yatim N.H. (2021). Linear shrinkage, strength and porosity of alumina-based ceramic foam with corn starch as pore former. Solid State Phenom..

[17] Jiang M.-Q., Jin X.-Y., Lu X.-Q., Chen Z.-L. (2010). Adsorption of Pb(II), Cd(II), Ni(II) and Cu(II) onto natural kaolinite clay. Desalination.

[18] Kweon D.-K., Choi J.-K., Kim E.-K., Lim S.-T. (2001). Adsorption of divalent metal ions by succinylated and oxidized corn starches. Carbohydr. Polym..

[19] Almohana A.I., Almojil S.F. (2025). Development of magnetic Kaolin/Starch/CuFe_2_O_4_/ZIF-9 composite for nickel adsorption: Physicochemical characterization and mechanistic insights. Colloids Surf. A Physicochem. Eng. Asp..

[20] Dermawan D., Karno S.I.C., Ramadani T.A., Widiana D.R., Ni’am A.C., Wang Y.-F., Setiawan A. (2025). Composite of kaolinite and activated carbon from pulp waste and sugarcane bagasse using one step KOH activation for rapid and highly efficient lead Pb(II) adsorption. Miner. Eng..

[21] Daou I., Lecomte-Nana G.L., Tessier-Doyen N., Peyratout C., Gonon M.F., Guinebretiere R. (2020). Probing the dehydroxylation of kaolinite and halloysite by in situ high temperature X-ray diffraction. Minerals.

[22] Hoang N.H. (2015). Nghiên cứu xác định nồng độ bromide và iodide trong nước vỉa dầu khí bằng phương pháp phân tích trắc quang. Petrovietnam J..

[23] Wolf E. (2018). Comparison of Methodologies of Using Colorimetry to Detect Low Concentrations of Bromide as a Hydrologic Tracer. https://digitalrepository.unm.edu/wr_sp/182/.

[24] Tran H.N. (2022). Improper estimation of thermodynamic parameters in adsorption studies with distribution coefficient KD (qe/Ce) or Freundlich constant (KF): Considerations from the derivation of dimensionless thermodynamic equilibrium constant and suggestions. Adsorpt. Sci. Technol..

[25] Thommes M., Kaneko K., Neimark A.V., Olivier J.P., Rodriguez-Reinoso F., Rouquerol J., Sing K.S. (2015). Physisorption of gases, with special reference to the evaluation of surface area and pore size distribution (IUPAC Technical Report). Pure Appl. Chem..

[26] Hug S.J., Sulzberger B. (1994). In situ Fourier transform infrared spectroscopic evidence for the formation of several different surface complexes of oxalate on TiO_2_ in the aqueous phase. Langmuir.

[27] Wijnja H., Schulthess C.P. (2000). Vibrational spectroscopy study of selenate and sulfate adsorption mechanisms on Fe and Al (hydr) oxide surfaces. J. Colloid Interface Sci..

[28] Al-Ghouti M.A., Khan M., Malik A., Khraisheh M., Hijazi D., Mohamed S., Alsorour S., Eltayeb R., Al Mahmoud F., Alahmad J. (2022). Development of novel nano-γ-Al_2_O_3_ adsorbent from waste aluminum foil for the removal of boron and bromide from aqueous solution. J. Water Process Eng..

[29] Liu S., Cheng G., Xiong Y., Ding Y., Luo X. (2020). Adsorption of low concentrations of bromide ions from water by cellulose-based beads modified with TEMPO-mediated oxidation and Fe(III) complexation. J. Hazard. Mater..

[30] El-Nemr M.A., El Nemr A., Hassaan M.A., Ragab S., Tedone L., De Mastro G., Pantaleo A. (2022). Microporous activated carbon from *Pisum sativum* pods using various activation methods and tested for adsorption of acid orange 7 dye from water. Molecules.

[31] Li Y., Zhao L., Lin L., Li E., Cao Q., Wei C. (2022). Relationships between X-ray diffraction peaks, molecular components, and heat properties of C-type starches from different sweet potato varieties. Molecules.

[32] Kosmulski M. (2023). The pH dependent surface charging and points of zero charge. X. Update. Adv. Colloid Interface Sci..

[33] Zhang Y.-Q., Wu Q.-P., Zhang J.-M., Yang X.-H. (2015). Removal of bromide and bromate from drinking water using granular activated carbon. J. Water Health.

[34] Ding L., Deng H., Wu C., Han X. (2012). Affecting factors, equilibrium, kinetics and thermodynamics of bromide removal from aqueous solutions by MIEX resin. Chem. Eng. J..

[35] Ho Y.-S., McKay G. (1999). Pseudo-second order model for sorption processes. Process Biochem..

[36] Tchomgui-Kamga E., Audebrand N., Darchen A. (2013). Effect of co-existing ions during the preparation of alumina by electrolysis with aluminum soluble electrodes: Structure and defluoridation activity of electro-synthesized adsorbents. J. Hazard. Mater..

[37] Dadwhal M., Sahimi M., Tsotsis T.T. (2011). Adsorption isotherms of arsenic on conditioned layered double hydroxides in the presence of various competing ions. Ind. Eng. Chem. Res..

[38] Zhang Q., Wang Y., Han J., Liu H., Zhang H., Wu Z., Zhang S., Han W., Ye X. (2024). Insights into adsorption behavior and mechanism of Br^−^ onto nickel–aluminum layered double hydroxides intercalated with different inorganic anions. Langmuir.

[39] Foo K.Y., Hameed B.H. (2010). Insights into the modeling of adsorption isotherm systems. Chem. Eng. J..

[40] Dubinin M. (1960). The potential theory of adsorption of gases and vapors for adsorbents with energetically nonuniform surfaces. Chem. Rev..

[41] Shi M., Guo C., Li J., Li J., Zhang L., Wang X., Ju Y., Zheng J., Li X. (2017). Removal of bromide from water by adsorption on nanostructured δ-Bi_2_O_3_. J. Nanosci. Nanotechnol..

[42] Zhang X., Wang X., Gao J., Li J., Liang X., Shou Q. (2024). Preparation of Ag-Cu-MOF-74 and its bromine adsorption in simulated saline water. Chem. Eng. Process.–Process Intensif..

[43] Gong C., Zhang Z., Qian Q., Liu D., Cheng Y., Yuan G. (2013). Removal of bromide from water by adsorption on silver-loaded porous carbon spheres to prevent bromate formation. Chem. Eng. J..

