# Supplementary material for: Bromide Sequestration Using a Low-Cost Hybrid Composite of Calcium, Activated Carbon, Kaolin, and Starch
Source: Polymers (Basel). 2026 Oct 20;18(20):2586. doi: 10.3390/polym18202586 (PMC13708984; doi:10.3390/polym18202586)
Supplement: Supplementary file 1 [file polymers-18-02586-s001.zip › polymers-4519523-supplementary.pdf]

Supplementary

Table S1. Experimental conditions verified and used in bromide adsorption experiments.

Experiment	Initial concentration, C_0 (mg L^{-1})	Solution volume, V (L)	Adsorbent mass, m (g)	Adsorbent dosage, m/V (g L^{-1})	Variable investigated
Effect of pH	20	0.025	0.10	4	pH (2–10)
Adsorbent dosage	30	0.025	0.05–1.50	2–60	Adsorbent dosage
Contact time	30	0.025	0.10	4	0–120 min
Agitation speed	30	0.025	0.10	4	50–250 rpm
Initial bromide concentration	5–200	0.025	0.10	4	Initial concentration
Temperature	30	0.025	0.10	4	25–55°C
Coexisting ions	30	0.025	0.10	4	Ion type

Table S2. Proposed FTIR band assignments and changes after Br^- adsorption

Assignment	Before adsorption (cm^{-1})	After adsorption (cm^{-1})	Interpretation
O–H stretching	3431	3419	Shift and intensity change indicating modification of hydroxyl and hydration environments
Carbonyl-related vibration	1799	1799	No significant change
Carbonate asymmetric stretching	1424	1419	Change in the local environment of calcium- and carbonate-containing sites
Si–O–Si / Si–O–Al / C–O–C stretching	1031	1028	Possible involvement of aluminosilicate and oxygen-containing groups
Low-wavenumber lattice feature	—	615	New feature attributed to perturbation of mineral-lattice vibrations
Secondary low-wavenumber feature	—	495	Weak shoulder associated with changes in the inorganic framework

Table S3. Effect of coexisting ions on adsorption capacity (q_e) and removal efficiency (%R).

Coexisting ion	q_e	R %	Note
Control	6.800	90.667	No competition
Cl^-	6.613	88.167	Weak competition
NO_3^-	6.475	86.334	Moderate competition
CO_3^{2-}	6.200	82.667	carbonate effect
SO_4^{2-}	5.975	79.667	Strong competition
PO_4^{3-}	5.650	75.334	Strongest single-ion competition
Mixed ions	5.350	71.334	Matrix suppression
Range	5.35–6.80	71.33–90.67	

Table S4. Effect of operational parameters, including pH, agitation speed, initial concentration (C_0), adsorbent dosage, temperature, and contact time, on adsorption capacity (q_e) and removal efficiency (%R).

Experiment	Condition	q_e (mg g^{-1})	Removal (%)	q_e range (mg g^{-1})	Removal range (%)
**pH**	2	4.667	93.340	**1.803–4.867**	**36.067–97.336**
	3	4.800	96.004		
	4	4.834	96.670		
	5	4.867	97.336		
	6	3.468	69.366		
	7	2.136	42.727		
	8	2.003	40.063		
	9	1.870	37.399		
	10	1.803	36.067		
**Adsorbent dosage (g)**	0.05	9.1	60.667	**0.467–9.100**	**60.667–93.400**
	0.1	5.775	77		
	0.2	3.4	90.667		
	0.5	1.388	92.5		
	1	0.699	93.4		
	1.5	0.467	93.167		
**Contact time (min)**	0	2.5	33.334	**2.500–6.895**	**33.334–91.933**
	5	3.8	50.667		
	10	4.775	63.667		
	20	5.7	76		
	30	6.288	83.834		
	45	6.638	88.5		
	60	6.8	91.5		
	90	6.863	91.733		
	120	6.88	91.933		
**Agitation speed (rpm)**	50	5.95	79.334	**5.950–6.820**	**79.334–90.933**
	100	6.713	89.5		
	150	6.8	90.667		
	200	6.82	90.933		
	250	6.788	90.5		
**Initial concentration, C_0 (mg L^{-1})**	5	1.117	89.345	**1.117–39.997**	**79.994–93.784**
	10	2.234	89.345		
	15	3.217	85.793		
	20	4.401	88.013		
	25	5.584	89.345		
	30	7.034	93.784		
	50	10.968	87.746		
	72	15.29	84.942		
	100	21.157	84.629		

	150	30.947	82.525		
	200	39.997	79.994		
**Temperature (°C)**	25	6.743	89.9	**6.445–6.743**	**85.932–89.900**
	30	6.702	89.359		
	35	6.661	88.818		
	40	6.614	88.187		
	45	6.567	87.556		
	50	6.506	86.744		
	55	6.445	85.932		

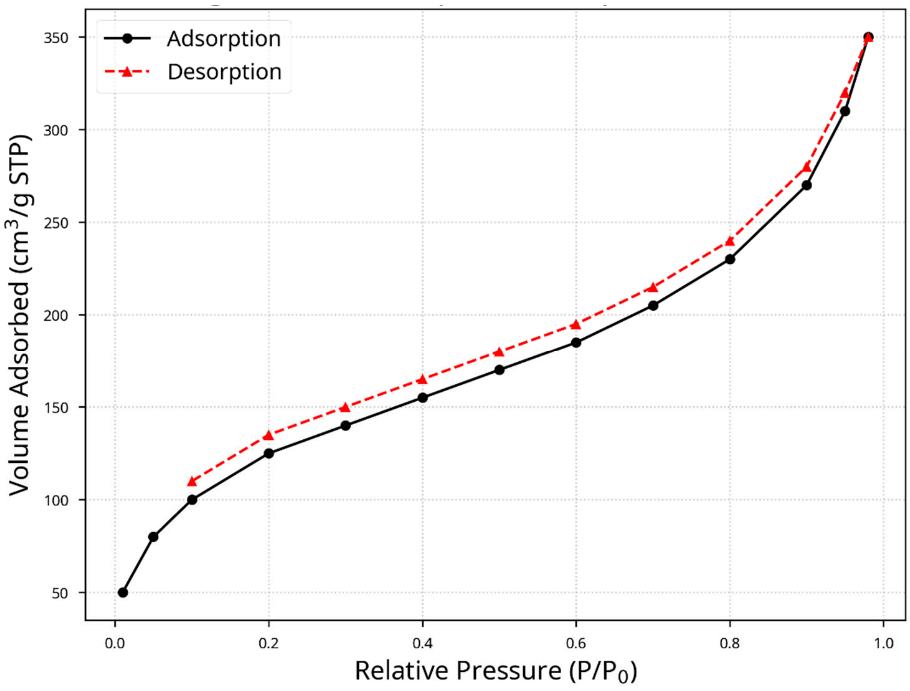

Figure S1. Nitrogen adsorption–desorption isotherm of the CAKS composite at 77 K, showing a Type IV profile with a distinct hysteresis loop, indicative of a predominantly mesoporous structure.

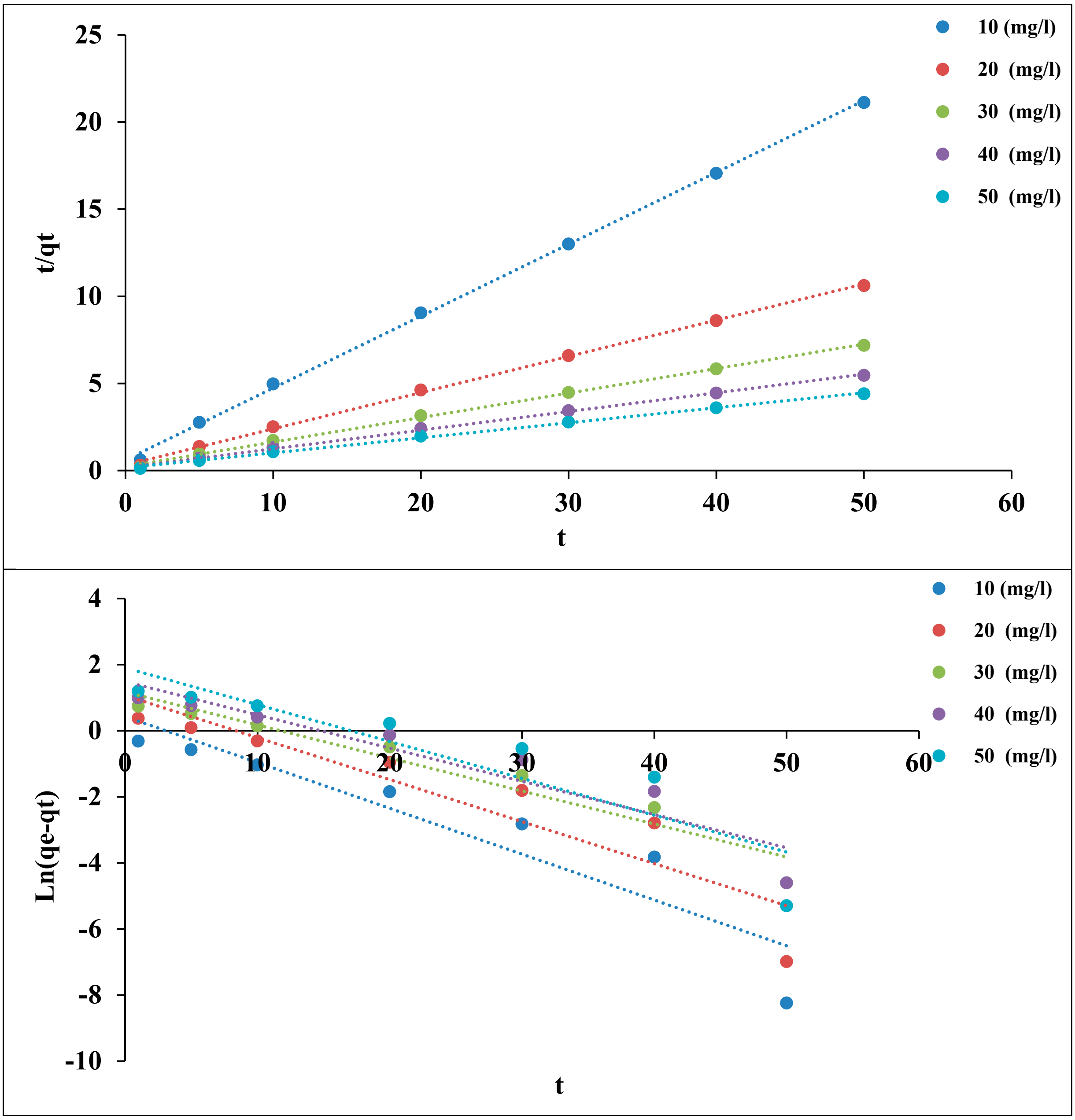

25
20
15
10
5
0
t/qt
0
10
20
30
40
50
60
t
10 (mg/l)
20 (mg/l)
30 (mg/l)
40 (mg/l)
50 (mg/l)
4
2
0
-2
-4
-6
-8
-10
Ln(qe-qt)
0
10
20
30
40
50
60
t
10 (mg/l)
20 (mg/l)
30 (mg/l)
40 (mg/l)
50 (mg/l)

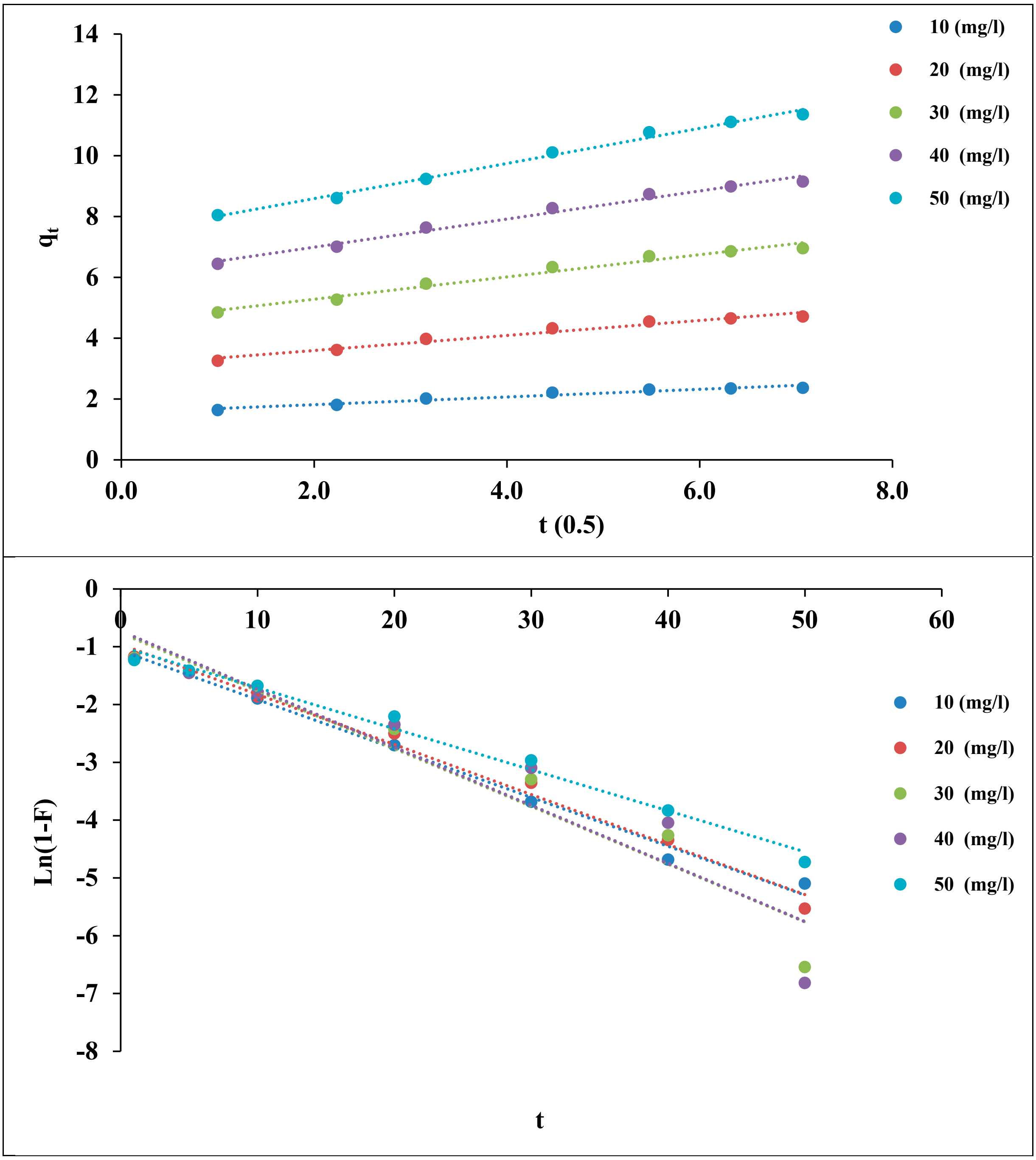

Figure S2. Kinetic analysis of bromide adsorption onto the CAKS composite at different initial concentrations: pseudo-first-order model, pseudo-second-order model, intraparticle-diffusion model, and film-diffusion model.
